# Global Environmental Geochemistry and Molecular Speciation of Heavy Metals in Soils and Groundwater from Abandoned Smelting Sites: Analysis of the Contamination Dynamics and Remediation Alternatives in Karst Settings

**DOI:** 10.3390/toxics13070608

**Published:** 2025-07-21

**Authors:** Hang Xu, Qiao Han, Muhammad Adnan, Mengfei Li, Mingshi Wang, Mingya Wang, Fengcheng Jiang, Xixi Feng

**Affiliations:** 1 College of Resource and Environment, Henan Polytechnic University, Jiaozuo 454003, China; xuhang689@126.com (H.X.); mingshiwang@hpu.edu.cn (M.W.); wangmy@hpu.edu.cn (M.W.); fc.jiang@hpu.edu.cn (F.J.); 10460230793@hpu.edu.cn (X.F.); 2 State Key Laboratory of Environmental Geochemistry, Institute of Geochemistry, Chinese Academy of Sciences, Guiyang 550081, China; 3 University of Chinese Academy of Sciences, Beijing 100049, China; 4 Henan Iron and Steel Group Co., Ltd., Zhengzhou 450046, China; mengfeili927@outlook.com

**Keywords:** molecular speciation, heavy metals, groundwater contamination, soil–groundwater interaction, environmental risk, remediation strategies

## Abstract

Abandoned smelting sites in karst terrain pose a serious environmental problem due to the complex relationship between specific hydrogeological elements and heavy metal contamination. This review combines work from across the globe to consider how karst-specific features (i.e., rapid underground drainage, high permeability, and carbonate mineralogy) influence the mobility, speciation, and bioavailability of “metallic” pollutants, such as Pb, Cd, Zn, and As. In some areas, such as Guizhou (China), the Cd content in the surface soil is as high as 23.36 mg/kg, indicating a regional risk. Molecular-scale analysis, such as synchrotron-based XAS, can elucidate the speciation forms that underlie toxicity and remediation potential. Additionally, we emphasize discrepancies between karst in Asia, Europe, and North America and synthesize cross-regional contamination events. The risk evaluation is complicated, particularly when dynamic flow systems and spatial heterogeneity are permanent, and deep models like DI-NCPI are required as a matter of course. The remediation is still dependent on the site; however, some technologies, such as phytoremediation, biosorption, and bioremediation, are promising if suitable geochemical and microbial conditions are present. This review presents a framework for integrating molecular data and hydrogeological concepts to inform the management of risk and sustainable remediation of legacy metal pollution in karst.

## 1. Introduction

Abandoned smelting areas, especially in karst regions, pose significant environmental challenges [1] due to the complex transport of heavy metals, including Pb, Cd, As, and Hg [2]. The fate and transport of these metals depend on site-specific geological, hydrological, and geochemical conditions [3], which are notably complex in karst areas with soluble carbonate rocks and erratic groundwater flow. However, the diversity of these environments adds complexity to the understanding and control of heavy metal pollution. It has been reported that these heavy metals in contaminated sites generally migrate along with soil colloids, which act as carriers and are absorbed by the groundwater system [4]. The spatial distribution of these metals is frequently associated with specific functional zones on smelting sites, while atmospheric deposition and surface runoff are held responsible for the contamination [5]. In karst areas, the rapid and dynamic process of groundwater flow can intensify the dissemination of pollutants, rendering the conventional risk assessment approach inadequate [6]. To remediate soil and groundwater contamination, approaches such as phytoremediation and the application of permeable reactive barriers have also been introduced; however, they are not always efficient, especially in areas where the hydrogeological conditions are complex [7]. Applications of these advanced modeling approaches and integrated risk assessment frameworks, for instance, the DI-NCPI model, may be able to improve the prediction and control of heavy metal pollution in such challenging settings [6]. Typically, an integrated mitigation model consisting of geochemical analysis, ecological risk assessment, and targeted remediation is crucial for controlling the ecological risks associated with abandoned smelting sites in karstic areas [8,9,10].

However, heavy metals, such as Pb, Cd, As, and Hg, have been well defined for their environmental persistence and systemic toxicity, which can cause severe impacts on human health even at low levels. Pb is highly neurotoxic, damaging cognitive function, and potentially contributing to neurodegenerative diseases such as Alzheimer’s and Parkinson’s [11]. Cd is known to be responsible for kidney trouble and skeletal injury, whereas As is a carcinogen to causes a variety of cancers [11,12]. Hg causes profound neurological deficits [13]. Environmental pollution of these metals is associated with industrialization, agricultural practices, and natural occurrences, leading to bioaccumulation in food chains, which further increases human exposure through the diet [14]. Characterization of their environmental speciation and transport processes is necessary in order to devise effective remediation strategies and public health interventions [11,12].

Given the hydrogeological and hydrochemical complexity associated with karst aquifers, risk assessments for contamination in these systems present several challenges. In contrast to granular or fractured aquifers, karst systems have fast transport mechanisms, including enlarged conduits and fractures, which spread contaminants rapidly and widely, often without the intervention of natural attenuation factors [15,16]. This rapid transportation becomes more severe during rainfall, when flow velocities and hydraulic heads can increase remarkably, and thus, stored pollutants can be flushed, inducing a change in hydraulic gradients and making it more challenging to control contaminants [15]. The carbonate mineralogy of karstic soils creates a specific geochemical context in which adsorption, co-precipitation, dissolution, and redox transformations are determining factors in metal speciation and mobility [17]. In abandoned smelting sites where legacy pollution continues to harm the environment, these interactions become significant; however, they are poorly described in karst environments [18]. Most conventional hydrogeological models fail to successfully reproduce the complex flow networks and mixing zones of a karst system, and there is an urgent need to develop models adapted to non-Darcian flows and the heterogeneous medium of karst aquifers [19,20]. Modeling approaches have advanced to represent the behavior of karst aquifers and their response to recharge events, enabling the investigation of contaminant transfer in this type of system both spatially and temporally [21,22]. It is well known that the source- and system-specificity of models and scale dependency make predictions uncertain, while the interpretation of numerical results is difficult because they are strongly affected by hydrological conditions and human impact [18]. Industry practice must change to accommodate karst’s inherent heterogeneity, spatially and temporally variable hydrologic conditions, and complex contaminant transport processes, requiring more sophisticated conceptual transport models and intensive fieldwork to obtain data that can be relied upon for specific site circumstances [17,19].

Advances in molecular-scale microanalytical methods, particularly X-ray absorption spectroscopy (XAS) and synchrotron-based techniques have significantly enhanced our ability to accurately determine heavy metal species in complex matrices, such as soils and sediments. These methods enable detailed speciation analysis of the metals and are crucial for understanding their bioavailability, systemic toxicity, and environmental persistence. For example, XAS can yield information about the oxidation state and local atomic environment of contaminants, which is essential for their mobility and reactivity and thus serves as a source of information to guide corrective remediation [23,24]. Techniques using synchrotron radiation, which provide high spatial resolution and high sensitivity, have revolutionized our understanding of metal interactions in natural systems by detecting metal speciation and its association with mineral and organic matter at the molecular scale [25,26]. Nevertheless, these similar approaches have been insufficiently applied in karst areas, with little comparative work, even though data have been compiled for abandoned smelting sites in diverse karst regions worldwide. These studies are interesting and important, as they play a crucial role in revealing geochemical patterns and contamination processes driven by geological and climatic differences at the regional scale [27,28]. The application of these advanced methods to karstic systems may enable an overall understanding of metal speciation and its environmental effects and derive generalizations that should be transferable to remediation. Therefore, such an approach will also address the current data scarcity issues and help devise site-specific solutions to heavy metal contamination in these vulnerable ecosystems [29,30].

Due to the dynamic nature of karst aquifers, which feature triple porosity (including rock matrix, fractures, and solutionally enlarged voids), it is challenging to model groundwater flow and contaminant transport, often resulting in underestimates of the risk of contamination [31]. For instance, in karst areas, heavy metal pollution (such as acid mine drainage (AMD)) poses health risks, as demonstrated in groundwater studies, which indicate that metals, including chromium and cadmium, exceed standards, posing particular danger to children [32,33]. The rapid recharge and lack of attenuation of contaminants in karst systems increase risks and necessitate specific remediation approaches to mitigate hazardous releases and protect groundwater supplies [34]. Understanding the geochemical fractionation and mobilization of heavy metals in this environment is crucial for effective eco-management and remediation practices [35]. Molecular speciation, which requires the accurate identification of the metal oxidation state and coordination environment at the atomic level, is unambiguously provided through the perhaps most popular approach, i.e., XAS. Using this approach, different chemical forms can be identified, which is crucial for studying the mobility, reactivity, and environmental hazards of contaminants [24]. Whereas chemical speciation groups elements in broader chemical forms without detailing them at the atomic level, it is concerned with operationally defined pools related to the mobility and bioavailability of the elements [36,37]. XAS, especially extended X-ray absorption fine structure (EXAFS) and X-ray absorption near-edge structure (XANES), can provide detailed information about the local chemical environment of metals, addressing effective remediation methods by elucidating how trace metals are sequestered in soils and other media [38,39].

Unlike previous reviews, which discuss only hydrogeological or chemical considerations in isolation, this review combines molecular-level metal speciation with karst hydrogeological complexity to deliver a mechanistic insight into contaminant migration and remediation. This concept thus represents a new tool to concentrate risk analysis and sustainable rehabilitation strategies in vulnerable karst contexts. This review integrates global information on environmental geochemistry and molecular speciation of heavy metal pollution at karst abandoned smelting sites. This demonstrates the importance of karst-specific hydrogeology and geochemistry in controlling the mobility, bioavailability, and relationships between metals and soil–groundwater. Through comparison between the datasets, the review revealed regional discrepancies as well as standard features of metal transport in karstic systems. It assesses the vulnerability of karstic aquifers on a molecular scale, associating speciation with ecological and human health threats. Finally, it considers strategic remediation in karst terrains to contribute to novel risk indices and mechanistically driven management approaches for sustainable remediation of contamination.

## 2. Materials and Methods

This review followed a systematic method for gathering, sifting, and synthesizing the literature about heavy metal contamination in karst areas. The exploration was conducted on the scientific databases Web of Science, Scopus, and Google Scholar, using keywords such as “karst hydrogeology,” “heavy metal speciation,” “abandoned smelting,” “groundwater contamination,” and “remediation strategies.” Only articles that were published in the last few decades were included. Studies on metal mobility, molecular speciation, interaction geochemistry, or remediation techniques in karst aquifers that were not peer-reviewed were not considered. Research that was neither related to karst systems and heavy metal pollution nor contained empirical or modeling data was excluded. When appropriate, qualitative and quantitative evidence was combined, and models or diagrams were created or recreated from the reported findings to complement the conceptual storyline of the review.

## 3. Vulnerability and Contamination Potential of Karst Groundwater

### New Horizons in Karst Remediation

Karst aquifers, with their rapid flow rates and reduced natural attenuation capacities, are particularly susceptible to contamination. Approximately 15% of ice-free terrestrial land is composed of carbonate bedrock, supplying more than 25% of the world’s population with fresh water from karst aquifers (Figure S1) [40]. Therefore, we need to reconsider risk assessment frameworks that incorporate molecular speciation data, along with hydrogeological attributes specific to these systems. The rapid transport of contaminants in the karst aquifers of the Tafna River Basin (western Algeria) highlights the importance of integrated planning with environmental risk assessment to preserve groundwater quality against urban development and industrial growth [41]. Open karst is highly permeable, allowing for the rapid transport of contaminants, such as emerging pollutants like microplastics, the behavior of which is still poorly understood in karstic environments [42]. The vulnerability of karst aquifers, for example, the Edwards Aquifer in Texas, is controlled by spatial and depth-specific gradients along the flow path, and shallow and unconfined areas are more vulnerable to pollutants [43]. EPIK and PI, both methodologies for vulnerability assessment, are developed to address the specific characteristics of the karst system. Around the world, karst aquifers are a crucial source of water for approximately one quarter of the world’s population, and therefore vulnerable to qualitative shocks, including the emergence of organic contaminants (EOCs) such as pharmaceuticals and pesticides at levels exceeding 100 ng/L [44]. The Edwards Aquifer, with an average flow rate that only occurs in response to events, also encounters contamination problems with harmful constituents, including nitrates and atrazine, based on past research indicating a lack of effective management approaches [45]. Furthermore, these vulnerabilities are exacerbated by climate change, which alters hydrological cycles and may increase the risk of contamination during specific periods [46].

However, the accuracy and validation of results are a problem [47]. Most of the heavy metal pollution in karst basins, such as the Sidi River, is caused by mining, and the metal concentration varies significantly due to the karstic aquifers’ specific features [33]. The hydrological dynamics of karst aquifers, such as turbulent flow and rapid infiltration, introduce significant complexity to traditional remediation methodologies, and as a result, the development of comprehensive geochemical models is necessary [16]. Human activities (e.g., agriculture and industry) accelerate the susceptibility of karst aquifers, as observed in northern China, where the quality of groundwater has deteriorated as a result of overpumping and pollution [48]. In consideration of its origin and dynamics, the hydrogeochemical features of karst groundwater are driven by the water–rock interaction and meteoric precipitation processes for risk evaluation [49]. As studied in the Pudding karst critical zone, the bioavailable and potentially ecological risks of heavy metals in karst soils suggest the importance of a comprehensive risk assessment that integrates both the speciation and mobility of contaminants [50]. The widespread sensitivity of karst aquifers to contamination should be considered to ensure comprehensive protection of groundwater. This can be achieved by combining molecular speciation data with a forensic interpretation of the hydrogeology and geochemistry of these aquifers to improve ecological and human risk assessments [51].

**Figure 1:**
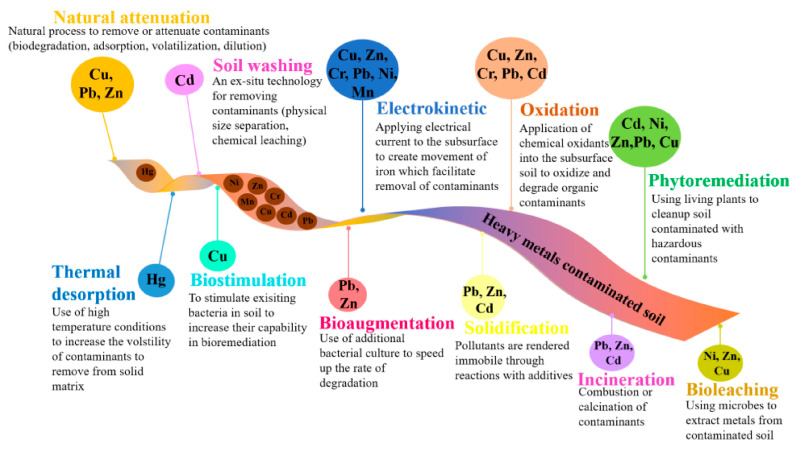
Strategies for heavy metal decontamination. Reprinted from [53], copyright (2023), with permission from the publisher.

Heavy metal pollution in karst regions is particularly challenging to remediate due to the complexity of karst hydrogeology. Karst aquifers are highly heterogeneous and anisotropic, and water and contaminants migrate primarily through conduits and fractures, characterized by rapid flow velocities but low filtration rates, making conventional remediation techniques (pump-and-treat and in situ chemical treatments) challenging to apply [15,16,52]. Consequently, soil remediation has become a crucial practice that contributes to the protection of both the environment and human health (Figure 1) [53]. The high velocity and limited retardation of pollutants in karst systems require versatile, multidisciplinary strategies that involve the study of hydrogeochemical properties and the molecular speciation of toxic metals [34,54]. For example, in some karsts, bioremediation has been effectively used when site-specific hydrological, geochemical, and microbial conditions are suitable, along with proper tracer application to determine regional contaminant pathways and residence times [55]. The US EPA’s concept of a technical impracticability (TI) waiver acknowledges the impossibility of meeting site closure levels at particularly challenging sites of this nature; however, TI waivers are rare due to the onerous nature of the process [52]. Case studies, for example, at the former Marietta Air Force Station, illustrate that a staged, multianalytical approach, including real-time analysis and geophysical methods, is required to define contamination and guide remediation activities [54]. Additionally, sustainable remediation technologies, such as phytoremediation and biosorption techniques, have been developed, which offer potential as eco-friendly and cost-effective alternatives to conventional methods, although their application within karst landscapes is site-specific [56]. Usually, the combination of sophisticated modeling tools and ambitious field surveys is essential in managing the karstic contamination of heavy metals [22]. Phytoremediation is known to be a cost-effective and sustainable strategy for the remediation of HM-contaminated soil by exploiting the natural ability of plants for HM bioaccumulation and stabilization [57,58]. Nonetheless, its efficiency is limited by the time required for remediation and the tolerance of plants to heavy metals, which can vary significantly among different plant species [59,60]. For example, some Compositae species exhibited differences in tolerance and accumulation of heavy metals, such as Cd, Pb, and Zn, in karst mine tailings [60]. In comparison, biosorption exhibits high specificity when targeting low-concentration pollutants but may be compromised in the presence of heterogeneous flow, as found in karst regions [57]. Traditional pump-and-treat systems may also be ineffective due to the rapid flow of water through conduits in karst, which can easily bypass treatment areas [57]. Hence, available remediation options, which have been designed for specific hydrogeological conditions, must be combined for effective management of heavy metals [61].

## 4. Karst Hydrogeology and the Transport of Heavy Metal

### 4.1. Specificities of Karst Systems: High Permeability and Varied Underground Drainage

**Figure 2:**
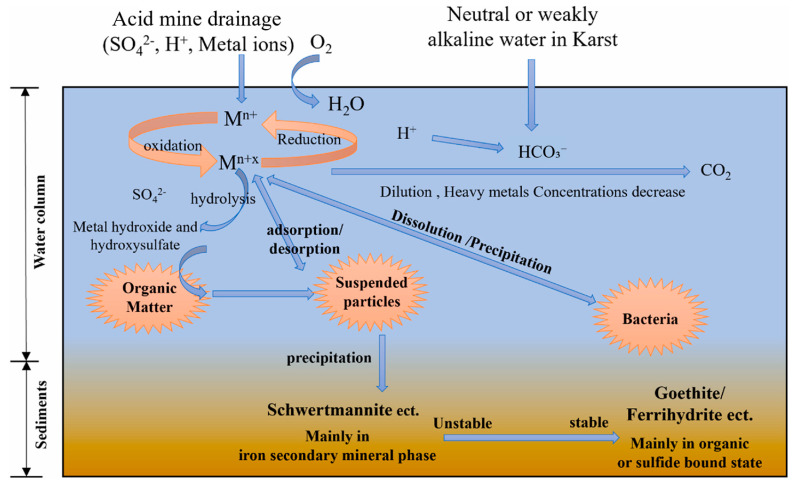
The schematic heavy metal distribution and migration in the karst mining area. Reprinted from [35], copyright (2024), with permission from the publisher.

The development of karst hydrogeology differs from that of any other groundwater system because it occurs in soluble carbonate rocks, such as limestone and dolomite, which have undergone extensive chemical weathering and dissolution. Soil carbonate above 5% controls the watershed’s dissolved inorganic carbon exports through carbonate weathering (Figure S2) [62]. This creates a heterogeneous subsurface consisting of interlocking conduits, fractures, caves, and sinkholes that form a patchwork of complex subsurface drainage networks. Characteristic traits of karst systems include extremely high permeability and hydraulic conductivity, primarily induced by the dissolution and enlargement of fractures and conduits, which result in rapid, often turbulent, groundwater flow through distinct conduits. Karst limestone has the highest hydraulic conductivity among other types of aquifers, as shown in Figure S3 [63]. This type of flow enables the rapid transport of water and solutes over great distances, in contrast to granular aquifers, where flow is diffusive and driven by matrix porosity [19,64,65]. The complex anisotropic flow condition of karst aquifers results in high spatial and temporal variability in the speed and direction of groundwater flow, which makes it challenging to predict contaminant transport and the corresponding modeling [15,66]. The existence of the epikarst zone, a high-weathered zone with a network of fissures and voids, also affects the recharge process and pollutant infiltration through selective flow paths that are detoured and bypassed the soil adsorption zones [16]. Complicating the situation, the flow dynamics can also be further exacerbated by hydrological variability, as storm events can quickly change flow conditions, escalating the possibilities of contaminant transport and the applicability of conventional aquifer remediation technologies [15,66]. As shown in Figure 2, metals enriched in AMD within the karst mine catchment are diluted and precipitated by neutral or weakly alkaline migrating waters as they travel, thereby lowering the concentration of metals and reducing their mobility [35]. This complexity has now been mainly captured and defined by numerical models, which have contributed to understanding how such systems work and how they evolve in space and time, such as in karstic aquifers [19,21]. The specific features of karst hydrogeological systems necessitate specific consideration in monitoring approaches, risk assessment and management of water resources and water quality to secure a further drinking water supply [67]. According to USEPA towards reducing the maximum contaminant level of As in drinking water to 10 μg/L, as presented in Table S1 [68,69].

### 4.2. Influence of Karst Hydrogeology on Heavy Metal Distribution and Mobility in Soil and Groundwater

**Figure 3:**
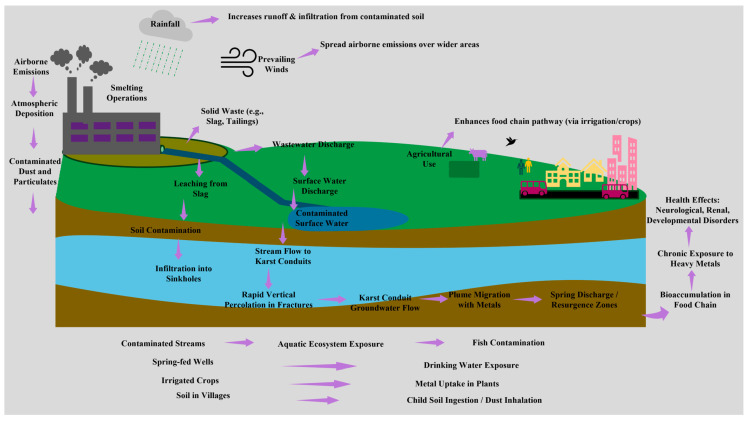
A conceptual model of heavy metal pollution pathways in karst areas. Smelting-related atmospheric emissions tend to lead to atmospheric and biotic deposition, soil and water pollution, and infiltration into the karst system. They are transported rapidly by fractures and conduits to involve both surface and groundwater. Exposure routes include irrigation and crop uptake, aquatic ecosystem disturbance, as well as human ingestion or inhalation, which can cause chronic health problems and lead to bioaccumulation in the food chain.

The special hydrogeological characteristics of the karstic system play a predominant role in the migration and transportation of heavy metals from abandoned smelting sites. These systems are highly susceptible to contamination due to the rapid flow-path transport and low filtration, which enables contaminants to rapidly move from soil surfaces to the conduits of groundwater without any substantial natural attenuation [15,34]. According to the previous study, pollutants are transported from the source to the exposure (and then inhaled, absorbed, or ingested based on their characteristics) and to the land used for disposal [18]. The complex processes of attenuation and transport of heavy metals derived from industry in karst systems are also illustrated, with an emphasis on environmental exposure and the biological and ecological risks associated with the bioaccumulation and transfer of these contaminants through the food chain into allotments and aquifers (Figure 3). Due to the high hydraulic conductance and turbulent flow regime in karst aquifers, contaminants, including heavy metals, can be rapidly transported from the mud surface to seepage in karst environments, which may be directed and harmful to groundwater [16,18]. Significant conduit pathways and conduit systems are also found in karst aquifers, leading to narrow pathways and, thus, highly heterogeneous contaminant concentration distributions with sharp concentration gradients [15,16]; however, in karstic areas, drinking and household water sources often come from springs and wells (Figure S4) [15]. This complicates the classic sampling and risk analysis procedures, as pollutants can be quickly conveyed to the spring outlets, frequently bypassing natural filtration processes [16,18]. In the soils overlying karst aquifers, the continuous switching between vadose and phreatic zones due to dynamic water tables and episodic recharge events renders HM retention and remobilization even more complex [22]. Heavy metals can also be adsorbed onto carbonate minerals or organic materials in the soil pore, the binding phase of which can be disrupted by variations in pH, redox conditions, or hydraulic flow, resulting in a burst release into groundwater [4,70]. High flow velocities and turbulent flow in karst systems are not only capable of serving as vectors for hydrophobic contaminants [71,72]; they are also proven carriers of sediment-associated contaminants, such as heavy metals. The specific characteristics of karstic aquifers necessitate targeted interventions to mitigate metal-related risks and protect downstream water uses [33,34]. Hydraulic conductivities in karst are highly variable, with conduits transmitting water and contaminants very quickly, often without significant filtration, while the rock matrix serves as slower, more stable pathways for flow [15,21]. Field studies, such as those in Kentucky, demonstrate that storm-induced hydraulic head rises can alter flow gradients, causing contaminated water to enter fractures and potentially impact nearby wells [73]. Furthermore, studies on subterranean streams demonstrate that water originating from the epikarst is an essential part of runoff, and its contribution during rainstorms should be considered, necessitating specific management practices for rainwater in karst areas [74]. This complexity necessitates the use of advanced modeling tools to predict contaminant transport and implement efficient measures [75].

## 5. Soil–Groundwater Interactions in Karst Terrains: Heavy Metals Retention and Speciation

### 5.1. Retention and Transport of Heavy Metals at the Soil–Water Interface

The coupling of soil and groundwater in the karst area had an important effect on the migration and transformation of heavy metals (especially the abandoned smelting sites). CaCO_3_ bedrock, which allows the rapid seepage of contaminants along the crevasse/fissure and sinkhole pathways, minimizes the contact time with the soil and decreases the potential for adsorption [15,66]; though, the exact nature of organic matter in CaCO_3_ sequestration remains indeterminate [62]. The carbonate minerals in the soils may affect metal retention through surface complexation and ion exchange processes, as heavy metal ions can strongly complex with carbonate minerals or organic matter, which restricts the transfer of metal [76]. Nevertheless, the nature of episodic recharge and dynamic water tables characteristic of karst conditions can intermittently remobilize adsorbed metals, facilitating their delivery into the groundwater [66]. Colloids also make the migration of heavy metals even more complex as carriers for Cd, Zn, As, and Pb, which are the elements mediated by colloids, and they can be prone to leaching into underground water with the soil profile [4]. During storm response, rapid groundwater velocity may facilitate the migration of particulate metals, many of which are colloids or relatively large particles, making it even more challenging to predict their fate [72,77]. Moreover, pH and redox conditions can affect the geochemical behavior of heavy metals in karst environments, resulting in the precipitation or dissolution of metal phases, and in turn, the species of heavy metals become soluble or mobile [35]. Noted that soil parameters, including pH value, organic matter content, clay content, and the composition of carbonate minerals, play a crucial role in the sorption capability and mobility of heavy metals. For instance, the pH of the soil did not correlate with the amount of Hg in the plants and soils, indicating that pH is not the determining factor in the transfer of Hg [78]. In karst soils with carbonate-buffered soil solution, consideration of these properties should be included in site-specific risk assessments.

The combination of these factors implies that there is a feedback regulation at the soil–water interface, serving as both a sink and source for heavy metals between the sorption and solution phases, making it difficult to predict and manage contaminant transport in karst systems [70]. Understanding these processes is crucial for developing sustainable remediation solutions and preserving groundwater quality in karst areas [79]. The soil mineralogical composition is a key factor in the retention and mobilization of heavy metals; adsorption, complexation, and precipitation are the primary processes regulating heavy metal bioavailability [80]. The interplay between metal(loid)s and mineral-organic matter associations further complicates these processes, as these associations can also immobilize metals, thereby preventing their mobilization and, consequently, their bioavailability through various mechanisms, such as heterogeneous nucleation and diffusion [81]. This is particularly the case with colloidal particle functionality, which has been identified as an important vector for trace metals. Transport studies have suggested that soil properties and redox status can impact the pathway of metal leaching [82,83]. Knowledge of these interactions is essential for developing effective remediation strategies and enhancing risk assessments of metal toxicity in the soil [80,84].

### 5.2. Influence of Geochemical Processes on Heavy Metal Speciation in Karst Lands

**Figure 4:**
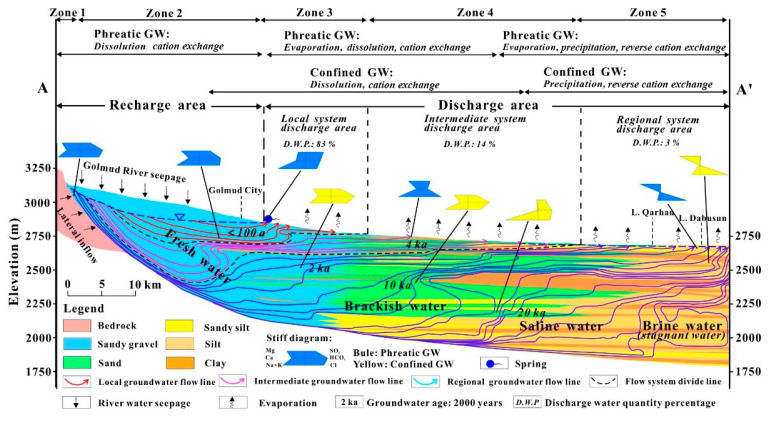
Conceptual model groundwater flow and hydrochemical evolution in the Golmud basin, China. Reprinted from [92], copyright (2017), with permission from the publisher.

Carbonate minerals of karstic systems have a significant impact on the geochemical processes controlling the speciation and transformation of heavy metals. Dissolution, precipitation and associated reactions with calcite and dolomite control the pH and alkalinity of karst waters and, thus, are important to the speciation and solubility of metals. High pH, characteristic of carbonate-rich conditions, decreases the solubility of some heavy metals by facilitating their precipitation as hydroxide, carbonate, or phosphate minerals, which effectively immobilizes them [85,86]. For example, calcite and dolomite have been indicated as good points to immobilize heavy metals such as Cu, Zn, Pb, Cd, Co, and Ni mainly through adsorption and precipitation processes in the experimental works, and the dolomite is generally more efficient for immobilization than calcite at similar pH (pH > 6.0) [85]. On the other hand, acidic or oxidizing conditions, which are common in acid mine drainage or during the decomposition of organic matter, can promote carbonate dissolution, leading to the mobilization of metals in the groundwater [87,88]. Adsorption on carbonate surfaces or secondary minerals, such as iron and manganese oxides, is also an important process controlling metal retention, as these materials offer active sites to which metals can be bound, thereby immobilizing them in less bioavailable conditions [89]. However, changes in redox state, ionic strength, and competing ions can affect adsorption equilibria, potentially leading to the desorption of the metal and increased mobility [89]. Synchrotron-based spectroscopic studies at the molecular scale have revealed the types of metals identified, which include surface complexes, precipitates, and co-precipitates with carbonate and oxide minerals [86]. These mechanisms are of great significance for mitigating heavy metal pollution in karst systems and should receive considerable attention for both control and management to maintain water quality in these vital aquifers [90,91]. According to the flowline distribution, the study region was divided into three groundwater flow systems: local, intermediate, and regional (Figure 4) [92].

The speciation and mobility of heavy metals in karst environments are significantly influenced by the dissolution of carbonate minerals, which buffer pH and regulate metal processes. Minerals of the carbon group, including calcite and dolomite, are dissolved, and cationic calcium, magnesium and bicarbonate increase alkalinity and pH triggering precipitation of metal carbonates and hydroxides accompanied by decreasing mobility of metal in solution through the transformation of dissolved metals into bioavailable in solid forms [85,93]. Oxidation–reduction reactions are also important, especially in the case of metallic species such as arsenic, where a change in redox state can appreciably affect solubility and toxicity [89]. Chemical equilibria are generally highly variable due to the dynamic hydrological conditions of a karst hydrological system, which are dominated by time-varying fluxes that cycle metals through dissolution, precipitation, adsorption, and desorption. For example, in dolomite aquifer systems, the initial pH and alkalinity of injected petroleum-produced water (PW) can control the removal of toxic metals, during which conditions, sorption and precipitation reactions take place for a prolonged period based on the alkalinity and pH that arise from dolomite dissolution [93]. According to Table S2, different filter methods were used for the removal of metal [94]. In addition, heavy metals can bind to organic ligands and carbonate species to form complexes, which, under certain environmental conditions, may have both immobilizing and mobilizing effects on the metal [35]. Sulfate-reducing bacteria can also contribute to removing metals by precipitating solid sulfide phases, especially at acidic pH [89]. Various heavy metals biosorption by different bacteria is shown in Table S3 [95,96,97,98,99,100,101,102,103,104,105]. Carbonate rocks can, however, naturally mitigate net acidity in acid mine drainage-affected karst settings, resulting in metals being concentrated in suspended solids and sediments [35]. A comprehensive understanding of these dynamic processes is crucial for more accurate assessment, prediction, and remediation of contamination in karst systems, as they control the fate, transport, and bioavailability of heavy metals in soils and groundwater [70].

## 6. Comparison of Heavy Metals Pollution in the Karst Areas on a Global Scale

### 6.1. Global Karst Landscapes and Heavy Metal Contamination at Abandoned Smelting Sites

It is an ideal site for studying the process of heavy metal mobility and contamination, particularly in relation to karst hydrogeology and geochemistry. In southwestern China, karst landscapes in regions such as Guizhou have been identified as highly vulnerable to heavy metal pollution from historical smelting and mining practices, with reported Cd content of up to 23.36 mg/kg in surface soil located at Pb-Zn mine areas [106]. The migration of heavy metals in these areas is controlled mainly by the karst environment and parameters (i.e., soil organic content, pH) competitive in the mobility and bioavailability of Cd, Pb, and Zn [70,106]. In the European karst areas, including Dalmatia, located by the Krka National Park (Croatia), the metal contamination of karst water is a fundamental issue, the industrial and municipal wastewater discharged being the origin of the contamination and Mn, Zn, and Fe representing the dissolved forms with high bioavailability [107]. The differences between the patterns of contamination in various karst regions are also evident in studies from the Sidi River karst basin in China, where metal concentrations in water bodies are strongly influenced by mine drainage and carbonate weathering [33,108]. The specific characteristics of karst aquifers (i.e., conduit development and hydrologic connections) can result in effects on the spatial distribution and migration of heavy metals (e.g., the polychromatic distribution of contaminants such as Cd, Zn, and Pb at smelting sites) [4]. Thus, these works emphasize the necessity of standardized datasets to pinpoint hotspots of contamination and trends, allowing for detailed analysis of how karst features control the fate of contaminants and guide specific remediation [35,109]. Conceptual model of Zn, Cd, Cu, and Pb sources and transport in Pb–Zn mine-impacted karst water system, as shown in Figure S5 [110].

**Figure 5:**
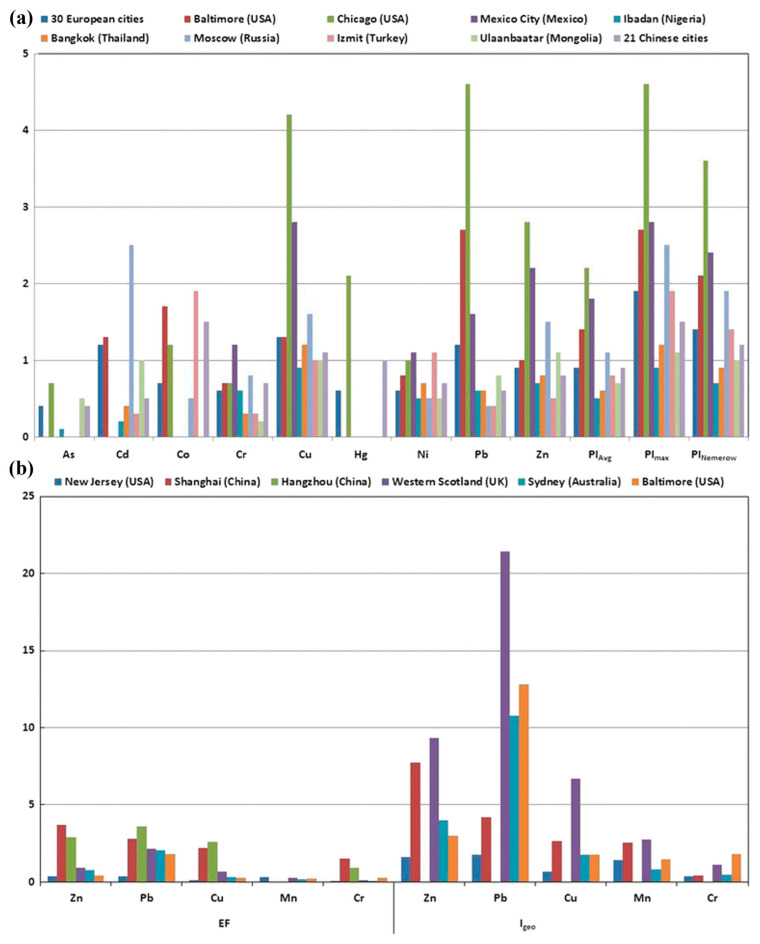
Heavy metal pollution indices of soils from selected world cities: (**a**) PI, PI_Avg_, PI_max_, and PI_Nemerow_, (**b**) Enrichment factor (EF) and Geo-accumulation index (Igeo). Reprinted from [111], copyright (2017), with permission from the publisher.

**Figure 6:**
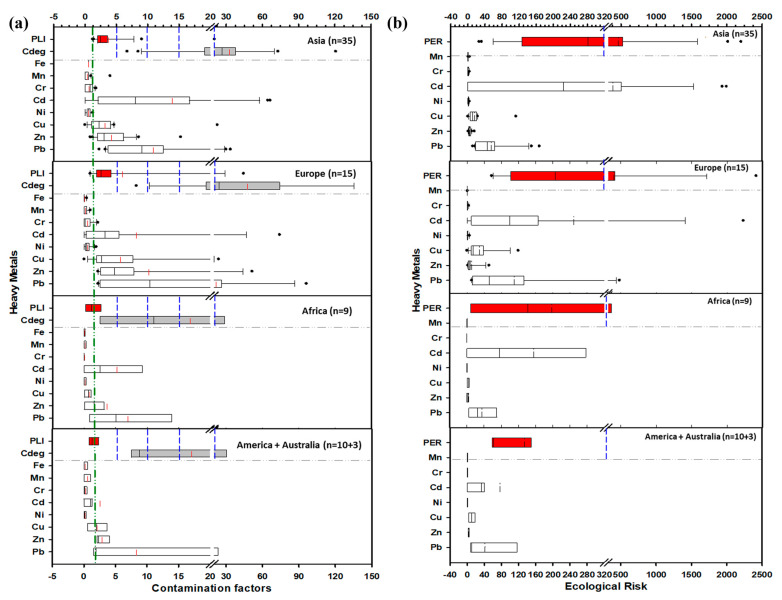
Exemplifies the continent-wide estimates derived from peer-reviewed data: (**a**) contamination degree (Cdeg) and pollution load index (PLI), and (**b**) ER and PER. Reprinted from [112], copyright (2022), with permission from the publisher.

Figure 5a presents PI, PI_Avg_, PI_max_, and PI_Nemerow_, demonstrating that European and American urban soils are moderately to significantly polluted by Pb, Cd, Cu, and Zn, which is higher than in cities in Africa and Asia; also Figure 5b presents the EF and Igeo factors of these urban soils [111]. Figure 6a presents box plots of the contamination factor, contamination degree (Cdeg), and pollution load index (PLI) for heavy metals in road dust worldwide, with a particular focus on pollution levels on a continent-by-continent basis; also, Figure 6b presents the box plots of ER and PER for the road dust heavy metals, which were used to display the ecological risk levels across continents [112]. Unfortunately, there is still no clear and comprehensive global dataset on heavy metal pollution in karst environments, which could be considered the first gap in research. Karst ecosystems are susceptible to human disturbance, and to design a disturbance index that encompasses the cultural, biotic, and hydrological changes in an area, it should be region-specific [113,114]. In addition, the ecological risk associated with pollutants such as Cd is geographically variable due to differences in species distribution and water quality characteristics, indicating that a “one-size-fits-all” strategy may not be applicable for water quality criteria [115]. This regional heterogeneity of ecological risk in wildlife underscores the need for location-specific management to address the local interaction between pollutants and wildlife in the unique environmental context of karst [116,117].

### 6.2. Comparative Hydrogeochemical Behavior and Metal Speciation in the Different Karsts

**Figure 7:**
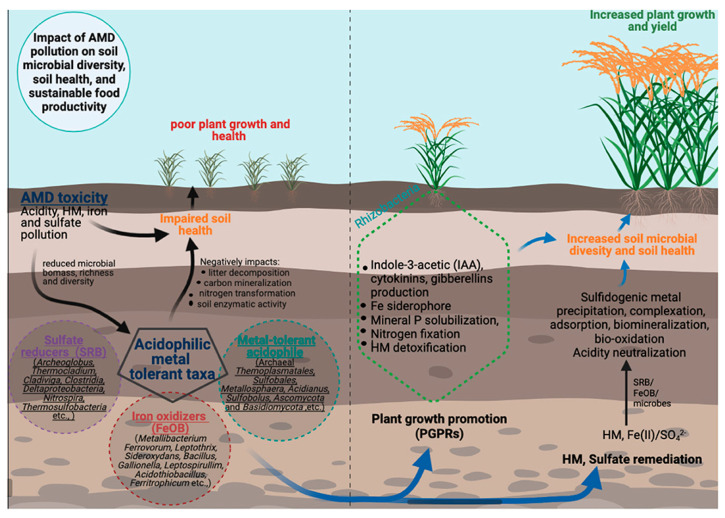
Long-term impact of acid mine drainage pollution on the health of agricultural soil, diversity, and function of microbes, and productivity of plants. The figure also demonstrates the promise of converting autochthonous microbial taxa (e.g., sulfate-reducing bacteria; iron-oxidizing bacteria) to benefit the health of AMD-impacted soils and microflora diversity for sustainable agro-systems. Reprinted from [118], copyright (2021), with permission from the publisher.

The comparison of heavy metals in karst areas at a global scale reveals standard features and specificities of different areas, which are strongly influenced by the local environment. Karst environments are particularly rich in the content of carbonate minerals and associated secondary precipitates, thus being able to constitute a primary buffering system for heavy metals in karst systems. This is reflected in the adsorption and precipitation mechanisms, where metals such as Cu, Zn, and Cd are precipitated by calcite and dolomite, particularly under basic conditions that promote metal precipitation and immobilization [85]. Nonetheless, differences in pH, redox potential, and organic matter content result in different metal speciation and mobility. For example, in southeast Asia, AMD-affected acid karst waters, metals remain more soluble and mobile, as evidenced by areas of the karst Xingren coalfield basin, where AMD dominates the hydrochemistry, characterized by high metal fluxes [35,87]. The various microbial communities and functions important for plant growth and productivity in AMD-contaminated agricultural soils are presented schematically in Figure 7 [118].

However, European karsts are also alkaline, and their filter function favors metal precipitation [85]. Hydrologic parameters, such as groundwater residence time and flow velocity, also influence contaminant dispersion patterns and dilution factors. Elevated groundwater velocities can promote the movement of particulate metals during storm events [77]. Through statistical comparison and the geospatial integration of these datasets, unique “fingerprints” of contamination can now be observed in each karst region, as evidenced by the example of the Lijiang River study, in which distinct sources of metals and relevant risks were identified through hierarchical clustering analysis [119]. This world comparative context emphasizes the need for locale-specific characterizations that integrate karst hydrogeology and molecular speciation data, as well as the development of generalized conceptual models transferable to karst-contaminated sites globally [70,120]. These evaluations are crucial for assessing ecological risk and informing remediation actions, particularly in areas where heavy metal pollution poses significant environmental and public health concerns [108,121].

## 7. Vulnerability Analysis and Risk Assessment of Heavy Metals of Karst Water Bodies

### 7.1. Susceptibility of Karst Aquifers to Heavy Metal Pollution

Due to its unique hydrogeological features, including the rapid flow of groundwater along extensive conduit passages and fractures, as well as the absence of natural attenuation, karst aquifers are highly sensitive to heavy metal pollution. These factors promote the rapid migration of contaminants, thereby circumventing the filtration and adsorption mechanisms that are standard in porous media aquifers for heavy metals [15,16]. This short flow path results in a shorter time for contaminants to contact certain reactive mineral surfaces or organic materials that would otherwise attenuate the mobility of the metal through sorption or precipitation [17]. The non-uniform characteristics of the karst feature lead to the tenuous spread of contaminant plumes, and local areas with high concentrations of metals also pose higher risks to the groundwater [18]. Dynamic hydrologic conditions, including recharge events and seasonal variations in water table elevations, have the potential to mobilize formerly sequestered metals, enhance their bioavailability, and complicate the application of traditional models for predicting contaminant fate [16]. This variability-rich environment requires specific monitoring methodologies to evaluate exposure [122] accurately. Karst aquifers are among the most vulnerable groundwater systems to legacy pollution from long-abandoned smelters, and a detailed investigation of contaminant transport paths and retention processes is required [18]. Several downstream hazard mapping procedures (EPIK, COP, etc.) have been developed, and conflicting results pose the question of validation and refinement of these techniques [47]. Rapid karst aquifer responses to precipitation also complicate the control of pollution, as pollutants may rapidly reach discharge points, causing a paradigm shift in the evaluation of vulnerability and attenuation of contaminants in karst groundwaters [17,122]. The incorporation of sophisticated modeling tools, including artificial neural networks and geographical information systems, enables refinement in the assessment of risk that better appreciates the complex hydrogeology of karst aquifers and potential sources of contamination from a range of anthropogenic activities [123,124]. Through understanding the known chemical forms of contaminants, we can help predict the mobility and bioavailability of contaminants, which will also inform advice on groundwater management and the unique role of karst regions in pollution indices [125,126]. This integrated approach not only contributes to risk analysis but also enables the taking of proactive actions aimed at reducing exposure, leading to significant benefits in terms of public health security in regions where karst aquifers are the sole sources of drinking water [123].

### 7.2. Correlation Between Molecular Speciation and Bioavailability and Risk in Karst System

**Figure 8:**
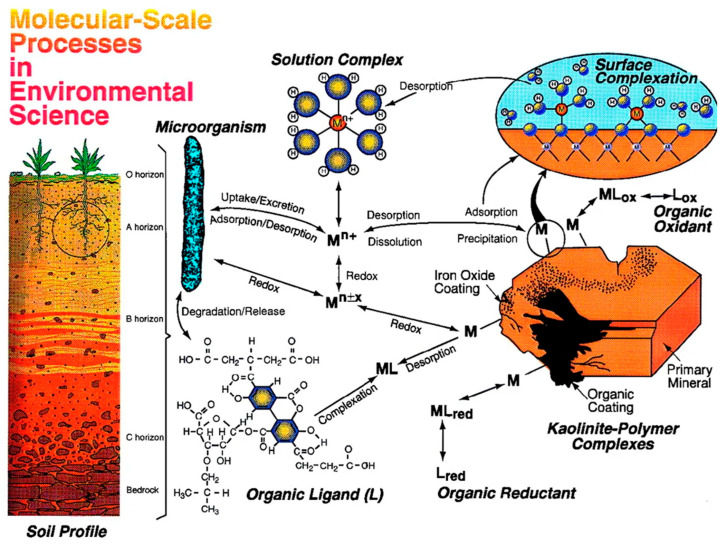
Molecular environmental processes for the fate of contaminants in soil and groundwater are portrayed schematically, modified from [29]. (Mineral Surfaces and Bioavailability of Heavy Metals: A Molecular-Scale Perspective. Proc. Natl. Acad. Sci. USA 1999, Copyright (1999) National Academy of Sciences, U.S.A.).

The molecular speciation of heavy metals is crucial in karst water for assessing their bioavailability and potential risks to human health and ecosystems. As shown in Table S4, the standards discharge of heavy metals, and their sources and public health impact [127]. The chemical forms of metals, including free ions, complexes, precipitates, or adsorbed species, which depend on molecular outer-sphere metal speciation, determine their mobility, toxicity, and bioavailability. For example, metals present in carbonate or sulfide insoluble forms are bioavailable only to a limited extent, while those as free ions or weakly adsorbed complexes are more readily available to organisms and able to bioaccumulate along food chains [29,128]. Recent developments in synchrotron-based spectroscopy and other molecular-scale spectroscopic methods have revealed various speciation patterns in karst groundwater, indicating complex interactions with mineral surfaces, redox gradients, and organic ligands [25]. Microbes play crucial roles in the degradation of organic contaminants and the transformation of heavy metals through redox reactions; in the case of vegetation, the (dotted circle in the soil profile, Figure 8) is an area of active chemical and biological activity where growing roots produce organic acids, sugars, and other components [29]. These speciation profiles can be site-specific, depending on geochemical factors and hydrological processes, and thus emphasize the importance of molecular data in ecosystem risk assessments [119,129]. It is well known that in the karst system, metals such as cadmium and lead exist in a bioavailable and relatively stable form that can pose significant risks to the ecology [119,121]. For elements like As or Pb, which are often present as adsorbed species on mineral surfaces and are therefore more bioavailable, techniques such as X-ray absorption fine structure (XAFS) spectroscopy have proven highly valuable in resolving their speciation [29]. Attaching speciation to bioavailability would enable risk assessments to proceed beyond total metal concentrations and support more informed predictions regarding exposure hazards by providing an avenue for the development of modified risk indices for karst aquifer systems. This methodology facilitates more informed management and remediation decisions in highly susceptible locations, considering the rapid movement of contaminants and variable geochemical states [130,131].

### 7.3. Ecotoxicological Effects of Heavy Metals in the Karst Ecosystems

**Figure 9:**
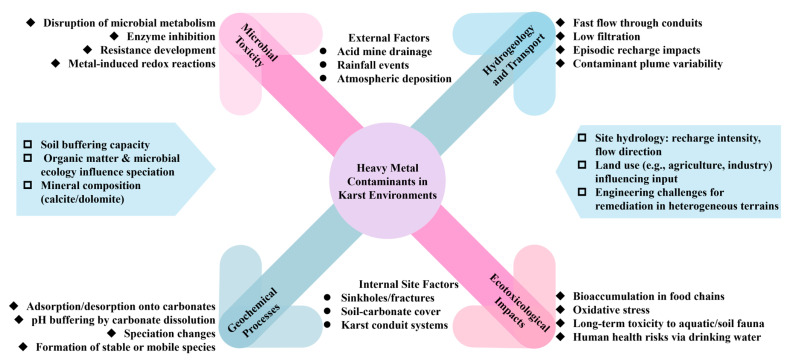
Schematic diagram representing how microorganisms, geochemistry, hydrogeology, and ecotoxicology control the heavy metal contamination process in karst systems.

A growing proportion of chemical substances is being generated globally in substantial volumes. Understanding their fate and ecotoxicological activity in the environment is crucial for making informed predictions and implementing suitable measures to mitigate detrimental impacts [132]. Additionally, it hinders crucial microbial functions, such as respiration and nitrification, which are essential for the biodegradation process [133,134]. As suggested by standardized toxicity tests, the sensitivity of microbes to heavy metals is generally higher than that of higher trophic levels, with EC50 values for microbes being generally much lower [133]. This collected body of literature highlights the requirement for detailed microbial toxicity data indicative of the level of dehydrogenase inhibition and depletion when assessing the effects of heavy metals on microbial community development in karst ecosystems [33,135]. A separate chapter could consolidate these findings and provide stronger emphasis on the issues of microbe vulnerability, while also guiding ecological risk assessments in these stressed environments [134,136]. Combining such ecotoxicological thresholds with molecular speciation data elucidates why sudden pH, carbonate buffering, or redox state changes can instantaneously mobilize bioavailable ions and drive community collapse. To address the information imbalance regarding microbiological and ecotoxicological responses to heavy metal contamination in karst environments, a synthesis of the various controlling and interacting mechanisms and regional factors is presented in Figure 9. The lack of specific quantitative microbial and ecotoxicological information about karst systems is a significant shortcoming in existing assessments. Future work should aim to establish adequate EC_50_/IC_50_ values for bacteria, algae, and invertebrates under karst-related conditions to inform risk characterization more effectively. While input data and concepts underlying these interacting drivers are illustrated in Figure 9, application of standardized toxicity thresholds would enhance predictive ecotoxicology for these sensitive landscapes.

Combining ecotoxicological thresholds with molecular speciation data is crucial for understanding how rapid changes in environmental parameters, such as pH and redox potential, can mobilize bioavailable ions, leading to the cessation of community function. In this context, gene diversity analysis can help achieve a comprehensive understanding. The biotic ligand model (BLM) also shows that metal toxicity is controlled by several factors in the environment (such as pH and the presence of competing ions) that influence metal binding to biological receptors [137]. In addition, the emergence of metal-resistant ecotypes complicates risk assessments, as these adaptations may alter the expected dose–response relationships in chemically contaminated sites [138]. Additionally, variations in pH and temperature can increase the bioavailability of harmful substances, such as saxitoxin, which may exacerbate the ecological risks associated with climate change [139]. The present synthesis emphasizes the importance of considering both chemical speciation and biological effects in ecotoxicological studies to enhance the prediction of ecosystem-level effects [140,141].

## 8. Conclusions

This review combines the intricate relationships between karst hydrogeology and molecular-scale heavy metal speciation to unravel the contamination processes at abandoned smelting sites worldwide. The unique rapid flow pathways and complex mineralogy of karst systems result in difficulties predicting contaminant transport and bioavailability, requiring a combined geochemical and hydrogeological approach. Global comparisons indicate that regionally distinct karst processes, climate, and anthropogenic history influence the mobility of heavy metals, but consistent indicators emerge in terms of carbonate-mediated metal partitioning and susceptibility to rapid transport due to low natural attenuation. Linking molecular speciation with bioavailability is crucial for making risk assessments to protect sensitive karst aquifers. The testing of remediation options highlights the need to develop interventions that are responsive to karst groundwater chemistry and utilize geochemical mechanisms for contaminant stabilization. Proceeding along, this holistic approach advances the karst-specific risk indices and remediation recommendations for managing the legacy smelting contamination in these ecologically and socioeconomically sensitive landscapes.

## Data Availability

Researchers wishing to access the data used in this study can make a request to the corresponding authors: hanqiao@hpu.edu.cn and adnan@mail.gyig.ac.cn.

## Supplementary Materials

The following supporting information can be downloaded at https://www.mdpi.com/article/10.3390/toxics13070608/s1. Figure S1 Outcrop of carbonate and evaporite rocks forming karst aquifers around the world; Figure S2 Global distribution of areas dominated by weathering of carbonate rocks/carbonate minerals [carbonate-rich soils (carbonate >5%) in non-carbonate rock and carbonate rock areas; Figure S3 Hydraulic conductivity (K) range for different types of rock; Figure S4 Sketch showing a generic karst aquifer with its most important subsystems; Figure S5 Conceptual model of the transfer of heavy metals in the karst system: (a) input with recharge water; (b) transfer combined with surface water–groundwater exchange; Table S1 Maximum permissible concentration of heavy metals for the drinking water quality (mg/L); Table S2 Based on different adsorbents in the removal of heavy metal contaminants; Table S3 Different heavy metal biosorption by different bacterial species; Table S4 Heavy metals discharge limits, sources, and public health impact.

## References

[1] Adnan M., Xiao B., Xiao P., Zhao P., Li R., Bibi S. (2022). Research Progress on Heavy Metals Pollution in the Soil of Smelting Sites in China. Toxics.

[2] Adnan M., Xiao B., Xiao P., Zhao P., Bibi S. (2022). Heavy Metal, Waste, COVID-19, and Rapid Industrialization in This Modern Era—Fit for Sustainable Future. Sustainability.

[3] Adnan M., Shao M., Ali M.U., Yan J., Xiao B., An X., Farooq M., Hayat K. (2025). Prospecting the engineered environmental carbon sinks and ensuring long-term sustainability of karst areas impacted by heavy metal. Sustain. Horiz..

[4] Liu J., Tang L., Peng Z., Gao W., Xiang C., Chen W., Jiang J., Guo J., Xue S. (2024). The heterogeneous distribution of heavy metal(loid)s at a smelting site and its potential implication on groundwater. Sci. Total Environ..

[5] Zhang Y., Li T., Guo Z., Xie H., Hu Z., Ran H., Li C., Jiang Z. (2023). Spatial heterogeneity and source apportionment of soil metal(loid)s in an abandoned lead/zinc smelter. J. Environ. Sci..

[6] Xue S., Wang Y., Jiang J., Tang L., Xie Y., Gao W., Tan X., Zeng J. (2024). Groundwater heavy metal(loid)s risk prediction based on topsoil contamination and aquifer vulnerability at a zinc smelting site. Environ. Pollut..

[7] Xue S., Ke W., Zeng J., Baltazar Tabelin C., Xie Y., Tang L., Xiang C., Jiang J. (2023). Pollution prediction for heavy metals in soil-groundwater systems at smelting sites. Chem. Eng. J..

[8] (2024). Fate and Transport of Heavy Metals in Soil, Surface Water, and Groundwater: Implications for Environmental Management. Int. J. Sci. Res. Manag. (IJSRM).

[9] Adnan M., Xiao B., Ali M.U., Xiao P., Zhao P., Wang H., Bibi S. (2024). Heavy metals pollution from smelting activities: A threat to soil and groundwater. Ecotoxicol. Environ. Saf..

[10] Bori J., Vallès B., Navarro A., Riva M.C. (2016). Geochemistry and environmental threats of soils surrounding an abandoned mercury mine. Environ. Sci. Pollut. Res..

[11] Tiwari B., Fatima G., Hadi N., Fedacko J., Magomedova A., Raza A.M., Alharis N., Qassam H., Alhmadi H.B., Parvez S. (2024). Metal Toxicity: Significant Health Assessment. Kufa Med. J..

[12] Abarikwu S.O. (2013). Lead, Arsenic, Cadmium, Mercury: Occurrence, Toxicity and Diseases. Pollutant Diseases, Remediation and Recycling.

[13] Rana M.N., Tangpong J., Rahman M.M. (2018). Toxicodynamics of lead, cadmium, mercury and arsenic-induced kidney toxicity and treatment strategy: A mini review. Toxicol. Rep..

[14] Rahman Z., Singh V.P. (2019). The relative impact of toxic heavy metals (THMs)(arsenic (As), cadmium (Cd), chromium (Cr)(VI), mercury (Hg), and lead (Pb)) on the total environment: An overview. Environ. Monit. Assess..

[15] White W.B. (2018). Contaminant Transport in Karst Aquifers: Systematics and Mechanisms. Karst Groundwater Contamination and Public Health: Beyond Case Studies.

[16] Field M. (1993). Karst Hydrology and Chemical Contamination. J. Environ. Syst..

[17] Sinreich M., Mudry J., Zwahlen F., Bertrand C., LaMoreaux J.W. (2014). Contaminant Attenuation in Karst Aquifers: A Paradigm Shift. H2Karst Research in Limestone Hydrogeology.

[18] Padilla I.Y., Vesper D.J. (2018). Fate, Transport, and Exposure of Emerging and Legacy Contaminants in Karst Systems: State of Knowledge and Uncertainty.

[19] Kaufmann G., Romanov D., Dreybrodt W., White W.B., Culver D.C., Pipan T. (2019). Chapter 86—Modeling the evolution of karst aquifers. Encyclopedia of Caves.

[20] Field M.S. (1997). RISK ASSESSMENT METHODOLOGY FOR KARST AQUIFERS: (2) SOLUTE-TRANSPORT MODELING. Environ. Monit. Assess..

[21] Kaufmann G., Romanov D., Dreybrodt W., White W.B., Culver D.C. (2012). Modeling of Karst Aquifers. Encyclopedia of Caves.

[22] Birk S. (2002). Characterisation of Karst Systems by Simulating Aquifer Genesis and Spring Responses: Model Development and Application to Gypsum Karst; Tübinger Geowissenschaftliche Arbeiten (TGA): Reihe C, Hydro- Ingenieur- und Umweltgeologie; 60.

[23] D’Amore J., Al-Abed S., Scheckel K., Ryan J.A. (2005). Methods for Speciation of Metals in Soils. J. Environ. Qual..

[24] Pickering I. (2003). X-Ray Absorption Spectroscopy as a Probe of Elemental Speciation.

[25] Brown G., Wang Y., Gelabert A., Ha J., Cismasu A., Ona-Nguema G., Benzerara K., Miot J., Menguy N., Morin G. (2008). Synchrotron X-ray studies of heavy metal mineral-microbe interactions. Mineral. Mag..

[26] McNear D.H., Tappero R., Sparks D.L. (2005). Shining Light on Metals in the Environment. Elements.

[27] Manceau A., Marcus M.A., Tamura N. (2002). Quantitative Speciation of Heavy Metals in Soils and Sediments by Synchrotron X-ray Techniques. Rev. Mineral. Geochem..

[28] Manceau A., Lanson B., Schlegel M., Musso M., Hazemann J.-L., Chateigner D., Lamble G. (2000). Quantitative Zn Speciation in Smelter Contaminated Soils By EXAFS Spectroscopy. Am. J. Sci..

[29] Brown G., Foster A., Ostergren J. (1999). Mineral Surfaces and Bioavailability of Heavy Metals: A Molecular-Scale Perspective. Proc. Natl. Acad. Sci. USA.

[30] Kirichkov M.V., Polyakov V.A., Shende S.S., Minkina T.M., Nevidomskaya D.G., Wong M.H., Bauer T.V., Shuvaeva V.A., Mandzhieva S.S., Tsitsuashvili V.S. (2024). Application of X-ray based modern instrumental techniques to determine the heavy metals in soils, minerals and organic media. Chemosphere.

[31] Kresic N. (2009). Foreword: Ground Water in Karst. Ground Water.

[32] Lan F.-N., Zhao Y., Li J., Zhu X.-Q. (2024). Health risk assessment of heavy metal pollution in groundwater of a karst basin, SW China. J. Groundw. Sci. Eng..

[33] Liao H.-W., Jiang Z.-C., Zhou H., Qin X.-Q., Huang Q.-B., Wu H.-Y. (2023). Heavy metal pollution and health risk assessment in karst basin around a lead-zinc mine. Huan Jing Ke Xue=Huanjing Kexue.

[34] Schindel G.M. (2017). Recommended strategies for the response to hazardous materials releases in karst. Karst Groundwater Contamination and Public Health: Beyond Case Studies.

[35] Qin S., Li X., Huang J., Li W., Wu P., Li Q., Li L. (2024). Inputs and transport of acid mine drainage-derived heavy metals in karst areas of Southwestern China. Environ. Pollut..

[36] Gaur A., Shrivastava B. (2012). A comparative study of the methods of speciation using X-ray absorption fine structure. Acta Phys. Pol. A.

[37] Tannazi F., Bunker G. (2005). Determination of chemical speciation by XAFS. Phys. Scr..

[38] Guillon E., Merdy P., Aplincourt M. (2003). Molecular scale speciation of first-row transition elements bound to ligneous material by using X-ray absorption spectroscopy. Chem.–A Eur. J..

[39] Porcaro F., Roudeau S., Carmona A., Ortega R. (2018). Advances in element speciation analysis of biomedical samples using synchrotron-based techniques. TrAC Trends Anal. Chem..

[40] Poeter E., Fan Y., Cherry J., Wood W., Mackay D. (2020). Groundwater in Our Water Cycle—Getting to Know Earth’s Most Important Fresh Water Source.

[41] Bensaoula F., Collignon B., Ali S., Armanuos A.M. (2023). Vulnerability to Pollution of Karstic Aquifers in the Tafna River Basin and Risk Mitigation Strategies (Northwest Algeria). Groundwater in Arid and Semi-Arid Areas: Monitoring, Assessment, Modelling, and Management.

[42] Campanale C., Losacco D., Triozzi M., Massarelli C., Uricchio V.F. (2022). An Overall Perspective for the Study of Emerging Contaminants in Karst Aquifers. Resources.

[43] Musgrove M., Jurgens B., Opsahl S. (2023). Karst Groundwater Vulnerability Determined by Modeled Age and Residence Time Tracers. Geophys. Res. Lett..

[44] Reberski J.L., Terzić J., Maurice L.D., Lapworth D.J. (2022). Emerging organic contaminants in karst groundwater: A global level assessment. J. Hydrol..

[45] Musgrove M., Katz B.G., Fahlquist L.S., Crandall C.A., Lindgren R.J. (2014). Factors affecting public-supply well vulnerability in two karst aquifers. Groundwater.

[46] Leins T., Scheller M., Çallı K.Ö., Ravbar N., Mayaud C., Petrič M., Liu Y., Hartmann A. (2025). A new process-based approach for defining karst aquifer vulnerability to contamination risks under global changes. Sci. Total Environ..

[47] Marín A.I., Andreo B., Stevanović Z. (2015). Vulnerability to Contamination of Karst Aquifers. Karst Aquifers—Characterization and Engineering.

[48] Zhang C., Zhang B., Zhang W., Zou J., Jia R., Yang Y. (2024). Hydrochemical Characteristics and Evolution under the Influence of Multiple Anthropogenic Activities in Karst Aquifers, Northern China. Water.

[49] Shan Q., Tian X., Xie H., Gong Z., Lin Y., Dang Z., Jun L., Zou S., Zhu T. (2024). Hydrogeochemical characteristics, driving factors, and health risk assessment of karst groundwater in Southwest Hubei Province, China. Water Environ. Res. Res. Publ. Water Environ. Fed..

[50] Zhang Q., Hah G.-L. (2022). Speciation Characteristics and Risk Assessment of Soil Heavy Metals from Puding Karst Critical Zone, Guizhou Province. Huan Jing Ke Xue=Huanjing Kexue/[Bian Ji Zhongguo Ke Xue Yuan Huan Jing Ke Xue Wei Yuan Hui "Huan Jing Ke Xue" Bian Ji Wei Yuan hui.].

[51] Wang S., Zhang C., Pei J. (2008). Research on the Vulnerability Assessment of Karst Groundwater. Groundwater.

[52] Field M.S. (2018). Investigating and Remediating Contaminated Karst Aquifers.

[53] Priya A.K., Muthiah M., Ali S.S., Kornaros M. (2023). Heavy Metals Removal from Contaminated Soil by Phytoremediation. Encyclopedia. https://encyclopedia.pub/entry/47036.

[54] Randrianarivelo M., Zhou W., Barsa M. (2019). Remedial investigations of karst aquifers: A case study at former Marietta Air Force Station, Lancaster County, Pennsylvania. Carbonates Evaporites.

[55] Byl T., Bradley M., Thomas L.K., Painter R. (2018). Bioremediation Potential in Karst Aquifers of Tennessee and Kentucky.

[56] Kumar H., Sahoo P.K., Mittal S., Shah M.P., Rodriguez Couto S., Kumar V. (2021). Chapter 23—Sustainable remediation of heavy metals: A review of current status and its future prospects. New Trends in Removal of Heavy Metals from Industrial Wastewater.

[57] Parmar S., Singh V. (2015). Phytoremediation approaches for heavy metal pollution: A review. J. Plant Sci. Res..

[58] Swetha N., Rajasekar B., Hudge B.V., Mishra P., Harshitha D.N. (2023). Phytoremediation of heavy metal contaminated soils using various flower and ornamentals. Int. J. Plant Soil Sci..

[59] Shakeel M., Yaseen T. (2015). An insight into phytoremediation of heavy metals from soil assisted by ancient fungi from glomeromycota-arbuscular mycorrhizal fungi. Sci. Technol. Dev..

[60] Zhu G., Zhao J., Chen Q., Guo Q., Cheng D., Bijaya G., Li W. (2022). The comparative potential of four compositae plants for phytoremediation of karst lead/zinc mine tailings contaminated soil. Bioresources.

[61] Sinha R.K., Herat S., Tandon P. (2003). A review of phytoremediation as a cost-effective, ecologically sustainable and socially acceptable bioengineering technlogy. National Environment Conference 2003.

[62] Shao M., Liu Z., Zeng S., Sun H., He H., Adnan M., Yan J., Shi L., Han Y., Lai C. (2025). Carbon sinks associated with biological carbon pump in karst surface waters: Progress, challenges, and prospects. Environ. Res..

[63] Kalhor K., Ghasemizadeh R., Rajic L., Alshawabkeh A. (2019). Assessment of groundwater quality and remediation in karst aquifers: A review. Groundw. Sustain. Dev..

[64] Fryar A.E., Mukherjee A., Scanlon B.R., Aureli A., Langan S., Guo H., McKenzie A.A. (2021). Chapter 2—Groundwater of carbonate aquifers. Global Groundwater.

[65] White W.B., White W.B., Culver D.C. (2012). Hydrogeology of Karst Aquifers. Encyclopedia of Caves.

[66] Ravbar N. (2013). Variability of groundwater flow and transport processes in karst under different hydrologic conditions. Acta Carsologica.

[67] Ponta G.M.L., Ponta G.M.L., Onac B.P. (2019). Karst Hydrogeology. Cave and Karst Systems of Romania.

[68] Ghosh S., Selvakumar G., Kennedy Ajilda A.A., Webster T.J., Shah M.P., Rodriguez Couto S., Kumar V. (2021). Chapter 10—Microbial biosorbents for heavy metal removal. New Trends in Removal of Heavy Metals from Industrial Wastewater.

[69] Adesiyan I., Bisi-Johnson M., Aladesanmi O., Okoh A., Ogunfowokan A. (2018). Concentrations and Human Health Risk of Heavy Metals in Rivers in Southwest Nigeria. J. Health Pollut..

[70] Zhang W., Xin C., Yu S. (2023). A Review of Heavy Metal Migration and Its Influencing Factors in Karst Groundwater, Northern and Southern China. Water.

[71] Mahler B.J., Personné J.-C., Lynch F.L., Van Metre P.C. (2004). Sediment and Sediment-Associated Contaminant Transport Through Karst.

[72] Mahler B.J., Personne J.-C., Lynch F.L., Van Metre P.C., Sasowsky I.D., Mylroie J. (2007). Sediment And Sediment-Associated Contaminant Transport Through Karst. Studies of Cave Sediments: Physical and Chemical Records of Paleoclimate.

[73] Polk J.S., Vanderhoff S., Groves C., Miller B., Bolster C. Complex Epikarst Hydrologeology and Contaminant Transport In A South-Central Kentucky Karst Landscape. Proceedings of the 16th International Congress of Speleology.

[74] Zhao H., Zhou H., Huang K., Pan Y., Peng Y., He X., Wang S., Wan J. (2024). Epikarst Controls of Runoff Composition in Subterranean Stream After Rainstorm Events. Hydrol. Process..

[75] Ghasemizadeh R., Hellweger F., Butscher C., Padilla I., Vesper D., Field M., Alshawabkeh A. (2012). Groundwater flow and transport modeling of karst aquifers, with particular reference to the North Coast Limestone aquifer system of Puerto Rico. Hydrogeol. J..

[76] Huang C., Elliott H., Ashmead R. (1977). Interfacial reactions and the fate of heavy metals in soil-water systems. J. Water Pollut. Control Fed..

[77] Vesper D.J., White W.B., Culver D.C. (2012). Contamination of Cave Waters by Heavy Metals. Encyclopedia of Caves.

[78] Şenilă M., Levei E.A., Şenilă L.R., Oprea G.M., Roman C.M. (2012). Mercury in soil and perennial plants in a mining-affected urban area from Northwestern Romania. J. Environ. Sci. Health Part A.

[79] Jiang J., Junlin C., Xiaoduo O., Haohao L., Wang S. (2024). Prediction of heavy metal contamination in soil-groundwater systems at contaminated sites. Environ. Technol..

[80] Sarkar S., Sarkar B., Basak B., Mandal S., Biswas B., Srivastava P. (2017). Soil mineralogical perspective on immobilization/mobilization of heavy metals. Adaptive Soil Management: From Theory to Practices.

[81] Li X., Schindler M., Zhou J., Samaradiwakara S., Wu L. (2025). Interaction between metal (loid) s and soil mineral-organic matter associations. Crit. Rev. Environ. Sci. Technol..

[82] Kretzschmar R., Schafer T. (2005). Metal retention and transport on colloidal particles in the environment. Elements.

[83] Löv Å. (2018). New Insights into Solubility Control Mechanisms and the Role of Particle-and Colloid-Facilitated Transport of Metals in Contaminated Soils.

[84] Sparks D.L. (2018). Kinetics and mechanisms of chemical reactions at the soil mineral/water interface. Soil Physical Chemistry.

[85] Savenko A.V. (2016). Experimental modeling of the immobilization of heavy metals at the carbonate adsorption–precipitation geochemical barrier. Geochem. Int..

[86] Salminen J., Kobylin P. (2006). Carbon Dioxide-Metal Carbonate Systems in Chemical Processes and Environmental Applications. ECS Meet. Abstr..

[87] Sun J., Tang C., Wu P., Liu C., Zhang R. (2013). Migration of Cu, Zn, Cd and As in epikarst water affected by acid mine drainage at a coalfield basin, Xingren, Southwest China. Environ. Earth Sci..

[88] Cidu R., Biddau R., Spano T. (2005). Temporal Variations in Water Chemistry at Abandoned Underground Mines Hosted in a Carbonate Environment. Mine Water Environ..

[89] Lee M.-K., Saunders J. (2003). Effects of pH on Metals Precipitation and Sorption: Field Bioremediation and Geochemical Modeling Approaches. Vadose Zone J..

[90] Özler H.M. (2010). Carbonate weathering and connate seawater influencing karst groundwaters in the Gevas–Gurpinar–Güzelsu basins, Turkey. Environ. Earth Sci..

[91] Li Vigni L., Daskalopoulou K., Calabrese S., Cardellini C., Kyriakopoulos K., Ionescu A., Brugnone F., Parello F., D’Alessandro W. (2019). Geochemical Characterization of Groundwater Quality in Hellenic Karst Systems.

[92] Xiao Y., Shao J., Frape S., Cui Y., Dang X., Wang S., Ji Y. (2018). Groundwater origin, flow regime and geochemical evolution in arid endorheic watersheds: A case study from the Qaidam Basin, Northwest China. Hydrol. Earth Syst. Sci. Discuss..

[93] Omar K., Vilcáez J. (2023). Transport of Ba, Sr, Cd, Pb, and as in dolomite saline aquifers injected with petroleum produced water. Geoenergy Sci. Eng..

[94] Vishnu D., Dhandapani B., Senthil Kumar K., Balaji G., Mahadevan S., Shah M.P., Rodriguez Couto S., Kumar V. (2021). Chapter 8—Removal of heavy metals from mine waters by natural zeolites. New Trends in Removal of Heavy Metals from Industrial Wastewater.

[95] Ansari M.I., Malik A. (2007). Biosorption of nickel and cadmium by metal resistant bacterial isolates from agricultural soil irrigated with industrial wastewater. Bioresour. Technol..

[96] Cristani M., Naccari C., Nostro A., Pizzimenti A., Trombetta D., Pizzimenti F. (2012). Possible use of Serratia marcescens in toxic metal biosorption (removal). Environ. Sci. Pollut. Res..

[97] Wu H., Wu Q., Wu G., Gu Q., Wei L., Kothe E. (2016). Cd-Resistant Strains of B. cereus S5 with Endurance Capacity and Their Capacities for Cadmium Removal from Cadmium-Polluted Water. PLoS ONE.

[98] Ozdemir S., Kilinc E., Celik K.S., Okumus V., Soylak M. (2017). Simultaneous preconcentrations of Co^2+^, Cr^6+^, Hg^2+^ and Pb^2+^ ions by Bacillus altitudinis immobilized nanodiamond prior to their determinations in food samples by ICP-OES. Food Chem..

[99] Tajer-Mohammad-Ghazvini P., Kasra-Kermanshahi R., Nozad-Golikand A., Sadeghizadeh M., Ghorbanzadeh-Mashkani S., Dabbagh R. (2016). Cobalt separation by Alphaproteobacterium MTB-KTN90: Magnetotactic bacteria in bioremediation. Bioprocess Biosyst. Eng..

[100] Yalçın M.S., Özdemir S., Kılınç E. (2018). Preconcentrations of Ni(II) and Co(II) by using immobilized thermophilic Geobacillus stearothermophilus SO-20 before ICP-OES determinations. Food Chem..

[101] Zárate A.M., Florez J.Z., Angulo E., Varela-Prieto L., Infante C., Barrios F., Barraza B., Gallardo D., Valdés J. (2017). Burkholderia tropica as a Potential Microalgal Growth-Promoting Bacterium in the Biosorption of Mercury from Aqueous Solutions. J. Microbiol. Biotechnol..

[102] Mondal P., Majumder C., Mohanty B. (2008). Treatment of arsenic contaminated water in a batch reactor by using Ralstonia eutropha MTCC 2487 and granular activated carbon. J. Hazard. Mater..

[103] Prasad K.S., Ramanathan A.L., Paul J., Subramanian V., Prasad R. (2013). Biosorption of arsenite (As^+3^) and arsenate (As^+5^) from aqueous solution by Arthrobacter sp biomass. Environ. Technol..

[104] Podder M., Majumder C. (2016). Corynebacterium glutamicum MTCC 2745 immobilized on granular activated carbon/MnFe2O4 composite: A novel biosorbent for removal of As(III) and As(V) ions. Spectrochim. Acta Part A Mol. Biomol. Spectrosc..

[105] Asadi Haris S., Altowayti W.A.H., Ibrahim Z., Shahir S. (2018). Arsenic biosorption using pretreated biomass of psychrotolerant Yersinia sp. strain SOM-12D3 isolated from Svalbard, Arctic. Environ. Sci. Pollut. Res..

[106] Luo K., Liu H., Zhao Z., Long J., Li J., Jiang C., Rao C. (2019). Spatial Distribution and Migration of Cadmium in Contaminated Soils Associated with a Geochemical Anomaly: A Case Study in Southwestern China. Pol. J. Environ. Stud..

[107] Mijošek T., Kljaković-Gašpić Z., Kralj T., Valić D., Redžović Z., Šariri S., Karamatić I., Filipović Marijić V. (2023). Spatial and temporal variability of dissolved metal(loid)s in water of the karst ecosystem: Consequences of long-term exposure to wastewaters. Environ. Technol. Innov..

[108] Liao H.-W., Jiang Z.-C., Zhou H., Qin X.-Q., Huang Q.-B., Zhong L., Pu Z.-G. (2022). Dissolved Heavy Metal Pollution and Assessment of a Karst Basin around a Mine, Southwest China. Int. J. Environ. Res. Public Health.

[109] Li W., Zhu T., Yang H., Zhang C., Zou X. (2022). Distribution Characteristics and Risk Assessment of Heavy Metals in Soils of the Typical Karst and Non-Karst Areas. Land.

[110] Qin W., Han D., Song X., Liu S. (2021). Sources and migration of heavy metals in a karst water system under the threats of an abandoned Pb–Zn mine, Southwest China. Environ. Pollut..

[111] Weissmannová H.D., Pavlovský J. (2017). Indices of soil contamination by heavy metals—Methodology of calculation for pollution assessment (minireview). Environ. Monit. Assess..

[112] Roy S., Gupta S.K., Prakash J., Habib G., Kumar P. (2022). A global perspective of the current state of heavy metal contamination in road dust. Environ. Sci. Pollut. Res..

[113] Van Beynen P., Townsend K. (2005). A disturbance index for karst environments. Environ. Manag..

[114] Mazzei M., Parise M. (2017). On the implementation of environmental indices in karst. Karst Groundwater Contamination and Public Health: Beyond Case Studies.

[115] Ding R., Wei D., Wu Y., Liao Z., Lu Y., Chen Z., Gao H., Xu H., Hu H. (2024). Profound regional disparities shaping the ecological risk in surface waters: A case study on cadmium across China. J. Hazard. Mater..

[116] Brand J.A., Martin J.M., Michelangeli M., Thoré E.S., Sandoval-Herrera N., McCallum E.S., Szabo D., Callahan D.L., Clark T.D., Bertram M.G. (2025). Advancing the spatiotemporal dimension of wildlife–pollution interactions. Environ. Sci. Technol. Lett..

[117] Fritz S.A., Bininda-Emonds O.R., Purvis A. (2009). Geographical variation in predictors of mammalian extinction risk: Big is bad, but only in the tropics. Ecol. Lett..

[118] Munyai R., Ogola H.J.O., Modise D.M. (2021). Microbial Community Diversity Dynamics in Acid Mine Drainage and Acid Mine Drainage-Polluted Soils: Implication on Mining Water Irrigation Agricultural Sustainability. Front. Sustain. Food Syst..

[119] Xu D., Wang Y., Zhang R., Guo J., Zhang W., Yu K. (2016). Distribution, speciation, environmental risk, and source identification of heavy metals in surface sediments from the karst aquatic environment of the Lijiang River, Southwest China. Environ. Sci. Pollut. Res..

[120] Liu D., Tian C., Chen X., Zhang W., Zhang X., Wang Z., Xu D., Chang Y. (2023). Insights into karst groundwater hydrogeochemical characteristics and spatial evolution in the Jinan karst aquifer system, northern China. Water Supply.

[121] Chen X., Wu P., Liu H., Li X. (2023). Source apportionment of heavy metal(loid)s in sediments of a typical karst mountain drinking-water reservoir and the associated risk assessment based on chemical speciations. Environ. Geochem. Health.

[122] Hamdan I. (2016). Characterization of Groundwater Flow and Vulnerability Assessment of Karstic Aquifers—Development of a Travel Time Based Approach and Application to the Tanour and Rasoun Spring Catchment (Ajloun, NW-Jordan). Ph.D. Thesis.

[123] Henry H.F., Suk W.A. (2017). Public health and karst groundwater contamination: From multidisciplinary research to exposure prevention. Karst Groundwater Contamination and Public Health: Beyond Case Studies.

[124] Bo L., Yi-Fan Z., Bei-Bei Z., Xian-Qing W. (2018). A risk evaluation model for karst groundwater pollution based on geographic information system and artificial neural network applications. Environ. Earth Sci..

[125] Monneron-Gyurits M., Soubrand M., Joussein E., Courtin-Nomade A., Jubany I., Casas S., Bahí N., Faz A., Gabarrón M., Acosta J.A. (2020). Investigating the relationship between speciation and oral/lung bioaccessibility of a highly contaminated tailing: Contribution in health risk assessment. Environ. Sci. Pollut. Res..

[126] Veselic M. (1995). Groundwater pollution control in fractured and karstified rocks. Advanced Methods for Groundwater Pollution Control.

[127] Sherlala A.I.A., Raman A.A.A., Bello M.M., Asghar A. (2018). A review of the applications of organo-functionalized magnetic graphene oxide nanocomposites for heavy metal adsorption. Chemosphere.

[128] Reuther R. (1996). Geochemical Speciation: Does it Help to Assess and Engineer the Impact of Metals?. Geochemical Approaches to Environmental Engineering of Metals.

[129] Fytianos K. (2019). Speciation Analysis of Heavy Metals in Natural Waters: A Review. J. AOAC Int..

[130] Adamo P., Agrelli D., Zampella M., Caporale A. (2024). Chemical speciation to assess bioavailability, bioaccessibility, and geochemical forms of potentially toxic metals (PTMs) in polluted soils. Environmental Geochemistry.

[131] Hao Y., Miao X., Liu H., Miao D. (2021). The Variation of Heavy Metals Bioavailability in Sediments of Liujiang River Basin, SW China Associated to Their Speciations and Environmental Fluctuations, a Field Study in Typical Karstic River. Int. J. Environ. Res. Public Health.

[132] Strotmann U., Durand M.-J., Thouand G., Eberlein C., Heipieper H.J., Gartiser S., Pagga U. (2024). Microbiological toxicity tests using standardized ISO/OECD methods—Current state and outlook. Appl. Microbiol. Biotechnol..

[133] Shafiq M., Rehman Y. (2024). Mechanisms of Toxicity of Heavy Metals and the Microbial Strategies for their Mitigation: A Review. J. Microbiol. Mol. Genet..

[134] Hu Z.-X., Wu Z.-Y., Luo W.-Q., Xie Y.-Q. (2024). Content, Sources, and Ecological Risk Assessment of Heavy Metals in Soil of Typical Karst County. Huan Jing Ke Xue=Huanjing Kexue.

[135] Wang S., Fang L., Dapaah M.F., Niu Q., Cheng L. (2023). Bio-remediation of heavy metal-contaminated soil by microbial-induced carbonate precipitation (MICP)—A critical review. Sustainability.

[136] Helf K.L. Mercury and methylmercury in the South Central Kentucky Karst: Its transportation, accumulation, and potential effects on vulnerable biota. Proceedings of the 2003 National Cave and Karst Management Symposium.

[137] Allen H.E., Janssen C.R. (2006). Incorporating bioavailability into criteria for metals. Soil and Water Pollution Monitoring, Protection and Remediation.

[138] Morgan A.J., Kille P., Stürzenbaum S.R. (2007). Microevolution and ecotoxicology of metals in invertebrates. Environ. Sci. Technol..

[139] Roggatz C., Fletcher N., Benoit D., Algar A., Doroff A., Wright B., Wollenberg Valero K., Hardege J. (2019). Saxitoxin and tetrodotoxin bioavailability increases in future oceans. Nat. Clim. Change.

[140] Reichardt W. (1996). Ecotoxicity of certain heavy metals affecting bacteria-mediated biogeochemical pathways in sediments. Sediments and Toxic Substances: Environmental Effects and Ecotoxicity.

[141] Cairns J. (1992). The threshold problem in ecotoxicology. Ecotoxicology.

