# Supplementary material for: Global Environmental Geochemistry and Molecular Speciation of Heavy Metals in Soils and Groundwater from Abandoned Smelting Sites: Analysis of the Contamination Dynamics and Remediation Alternatives in Karst Settings
Source: Toxics. 2025 Jul 21;13(7):608. doi: 10.3390/toxics13070608 (PMC12300227; doi:10.3390/toxics13070608)
Supplement: Supplementary file 1 [file toxics-13-00608-s001.zip › toxics-3723256-supplementary.pdf]

Supplementary materials

Global environmental geochemistry and molecular speciation of heavy metals in soils and groundwater from abandoned smelting sites: Analysis of the contamination dynamics and remediation alternatives in karst settings

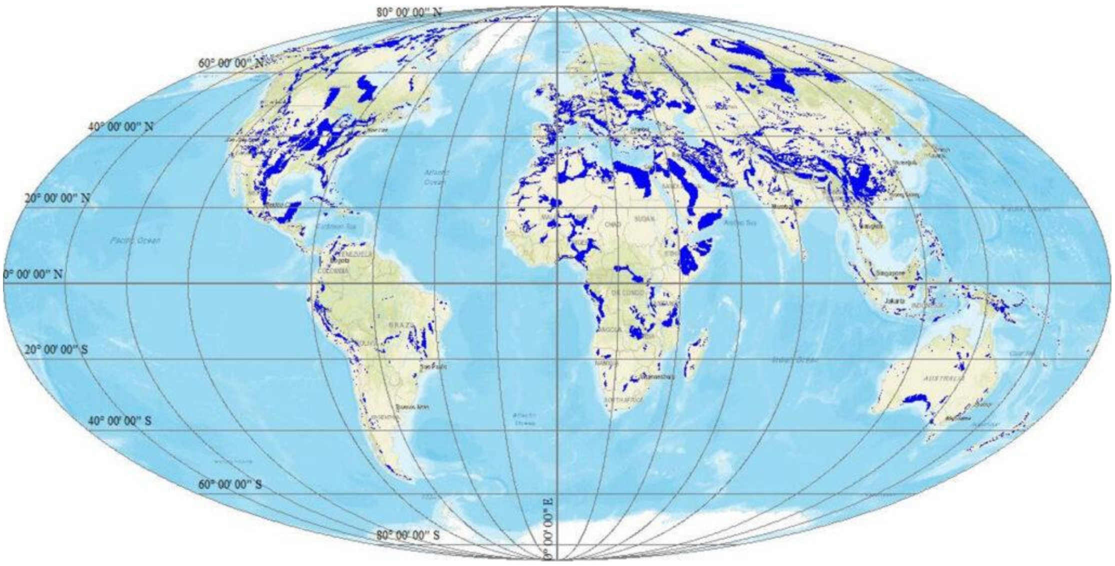

Figure S1 Outcrop of carbonate and evaporite rocks forming karst aquifers around the world. Reprinted from [1], copyright (2020), with permission from the publisher.

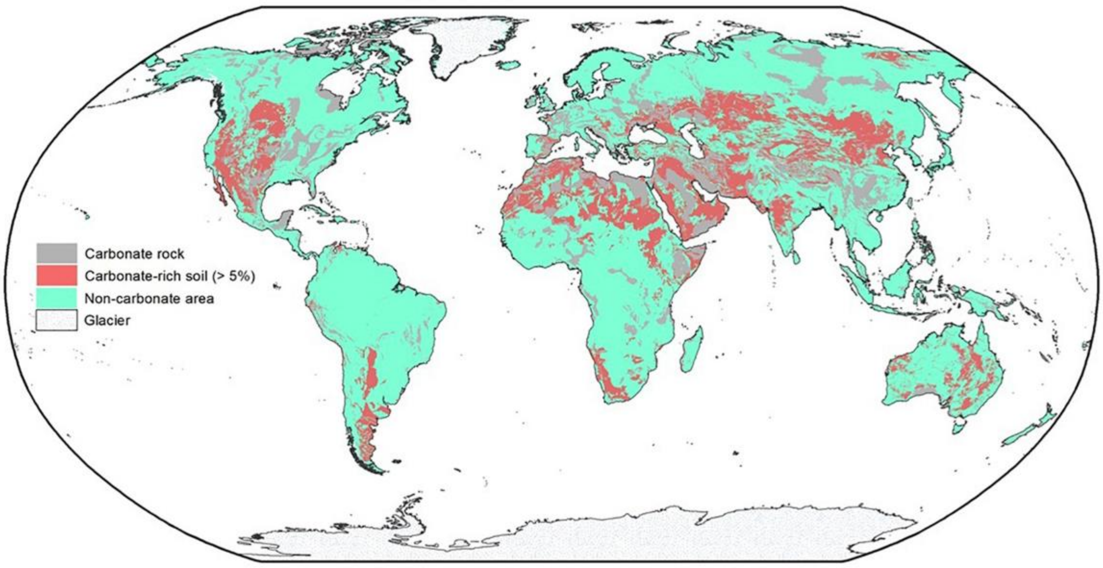

Figure S2 Global distribution of areas dominated by weathering of carbonate rocks/carbonate minerals [carbonate-rich soils (carbonate >5%) in non-carbonate rock and carbonate rock areas]. Preprinted from [2], copyright (2025), with permission from the publisher.

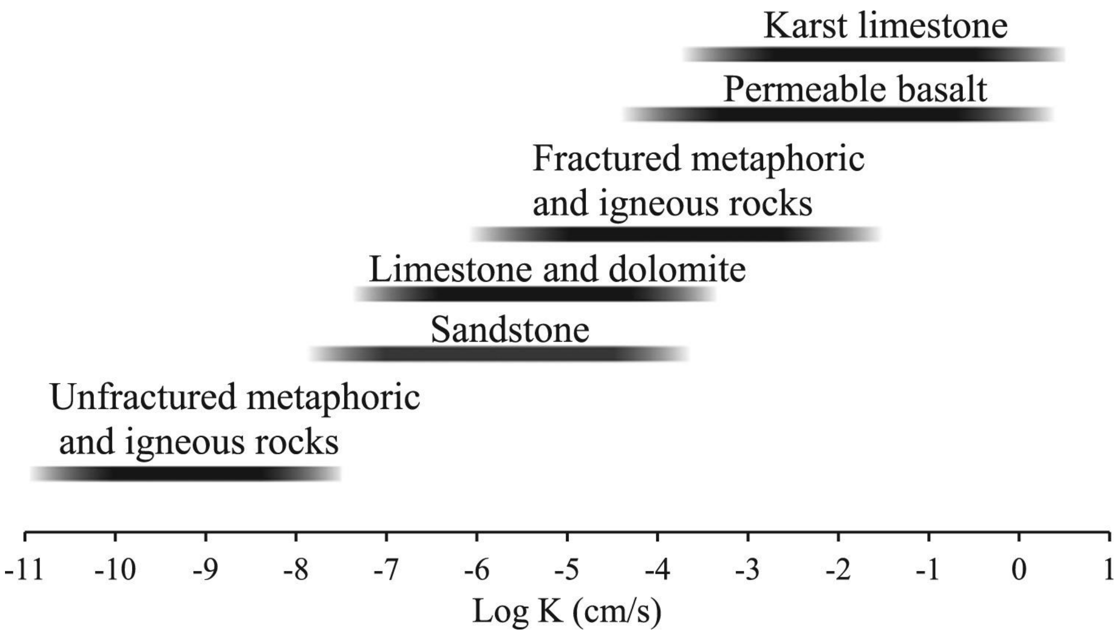

Figure S3 Hydraulic conductivity (K) range for different types of rock. Reprinted from [3], copyright (2019), with permission from the publisher.

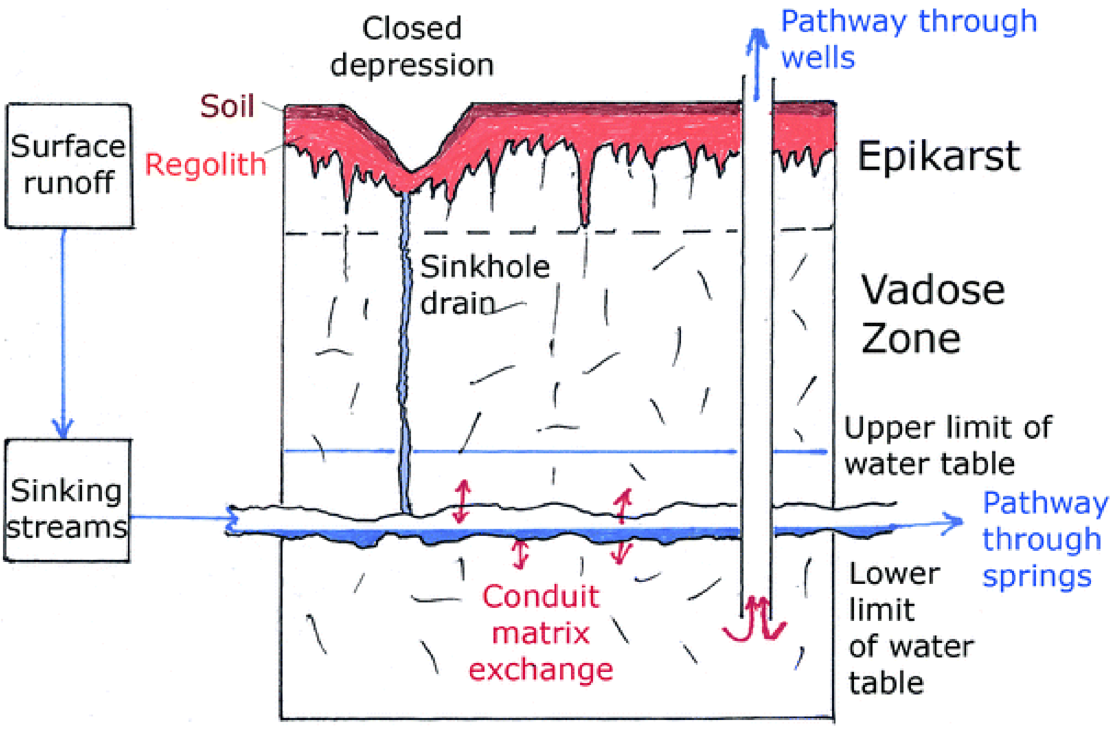

Figure S4 Sketch showing a generic karst aquifer with its most important subsystems. Reprinted from [4], copyright (2018), with permission from the publisher.

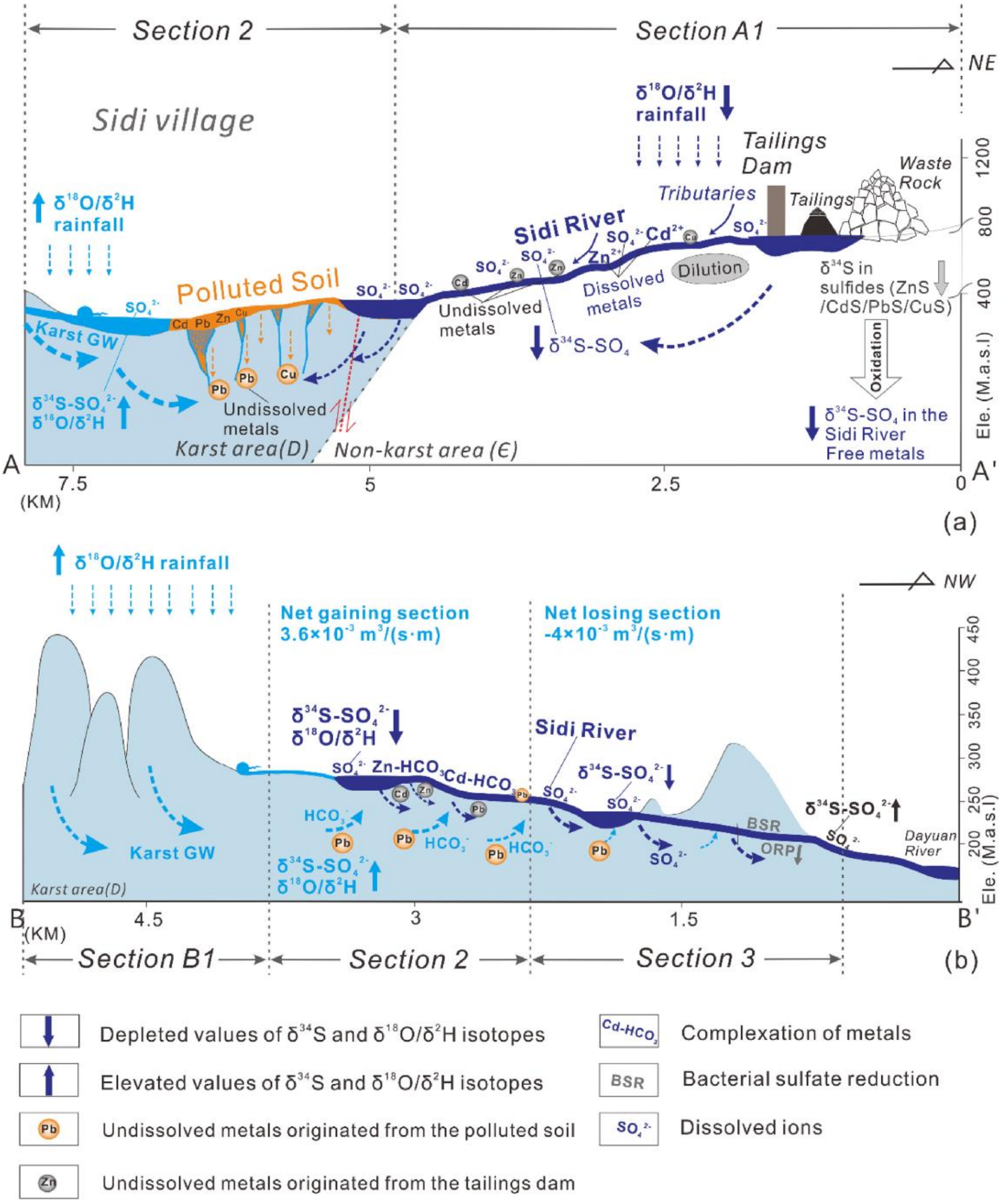

Figure S5 Conceptual model of the transfer of heavy metals in the karst system: (a) input with recharge water; (b) transfer combined with surface water–groundwater exchange. Reprinted from [5], copyright (2021), with permission from the publisher.

Table S1 Maximum permissible concentration of heavy metals for the drinking water quality (mg/L) [6, 7].

Element	EC (1998)	WHO (2004)	USEPA (2009)
Arsenic	0.01	0.01	0.010
Cadmium	0.005	0.003	0.005
Chromium	0.05	0.05	0.1
Cobalt	–	–	–
Copper	2	2	1.3
Iron	0.2	2	–
Lead	0.01	0.01	0.015
Manganese	0.05	0.4	0.05
Mercury	0.001	0.001	0.002
Nickel	0.02	0.02	–
Tin	–	[a]200	–
Vanadium	–	–	–

EC, European Commission; *WHO*, World Health Organization; *USEPA*, United States Environmental Protection Agency.

[a] mg/kg in food

Table S2 Based on different adsorbents in the removal of heavy metal contaminants [8].

Filter	Composition of filters and their properties	Metal removal
Fly ash	Fly ash prepared from peat fired station with the hydraulic conductivity of 1.3 m/day, porosity of 58%, and bulk density of 0.83 t/m^3	Used in the metal removal of zinc, lead, and cadmium
Calcareous shale	Rocks obtained were dried to the size of 10 mesh at the laboratory-scale level	Arsenic, lead, copper, and iron were removed
Limestone	Calcite limestones of the particle size that ranges from 0.42–0.59 mm to 0.045 µm, with the flow rate ranging from 1 to 10 mL/min	Removal of sulfate
Olivine	Derived from olivine dust, nonferrous,	Removal of copper
Materials from steel slag	Particle size between the range 24.5 and 24.1 µm	Removal of copper and lead

Zeolites	Clinoptilolite interchanged with iron material	Both batch and column studies were performed to remove the hazardous arsenic metal
Zeolites	Ash based on synthetic zeolites	Hazardous contaminants such as zinc, copper, nickel, iron, lead, barium, and cadmium are removed

Table S3 Different heavy metal biosorption by different bacterial species.

**Heavy**	**Bacterial**	**References**
Cd	Bacillus cereus, Pseudomonas spp., S. marcescens	[9-11]
Cr	*B. altitudinis, Serratia marcescens*	[10, 12]
Co	*Bacillus altitudinis, Geobacillus stearothermophilus, Alphaproteobacterium*	[12-14]
Ni	*Pseudomonas* spp., *G. stearothermophilus*	[9, 14]
Hg	*B. altitudinis, Burkholderia tropica*	[12, 15]
As	*Corynebacterium glutamicum, Arthrobacter* sp., *Ralstonia eutropha, Yersinia* sp.	[16-19]

Table S4 Heavy metals discharge limits, sources, and public health impact [20].

**Heavy metal**	**Maximum industrial effluent discharge standard (mg/L)**			**Health effects**	**Sources**
	**EPA**	**Malaysian (DOE)** **Standard A**	**Standard B**		
Pb	0.10	0.1	0.5	High risk of lower IQ and hyperactivity in Children May cause slow growth, hearing and anemia problems in children	Metal plating, mining activities, paint manufacture industries

				Causes cardiovascular effects, increased blood pressure and hypertension in adults	
As	0.01	0.05	0.1	Carcinogenic, dermatological, cardiorenal and gastrointestinal effects	Mining, smelting of arsenic-bearing minerals, pesticides, etc.
Ni	0.2	0.2	1	DNA damage, eczema, phyotototoxicity, respiratory cancer	Metal plating, mining, fertilizers, tanneries, batteries, paper, pesticides, electronics, petrochemical, textile
Cr	0.05[a] 0.10[b]	0.2	0.1	Kidney circulation, lung cancer, dermatitis	Plastic, pigment, wood preservative, electroplating, leather tanning, cement, mining, dyeing and fertilizer
Cu	0.25	0.2	1	Abdominal pain, diarrhea, weakness, cramps, diarrhea, etc.	Mining, refining ores, fertilizer industries, tanneries, batteries, paper industries
Zn	1	1	1	Abdominal pain, phytotoxic, anemia	Pharmaceuticals, galvanizing, paints, pigments, insecticides, cosmetics
Cd	0.01	0.01	0.02	Detrimental effects on kidney, lungs, liver, heart and bones of human being	Metal plating, mining activities, paint manufacture industries
Hg	0.05	0.005	0.05	Harmful effects on nervous, digestive and immune	Coal-fired power stations

				systems, lungs and kidneys<br><br>Corrosive to the skin, eyes and gastrointestinal tract, and may induce kidney toxicity if ingested	Residential coal burning for heating and cooking<br><br>Industrial processes<br><br>Waste incinerators<br><br>Mining for mercury, gold and other metals.

[a] Cr(VI)

[b] Cr(III)

References

1. Eileen Poeter, Y.F., John Cherry, Warren Wood, Doug Mackay, *Groundwater in our water cycle – getting to know Earth's most important fresh water source.* The Groundwater Project, Guelph, Ontario, Canada, 2020.
2. Shao, M., et al., *Carbon sinks associated with biological carbon pump in karst surface waters: Progress, challenges, and prospects.* Environmental Research, 2025. **267**: p. 120712.
3. Kalhor, K., et al., *Assessment of groundwater quality and remediation in karst aquifers: A review.* Groundwater for Sustainable Development, 2019. **8**: p. 104-121.
4. White, W.B. *Contaminant Transport in Karst Aquifers: Systematics and Mechanisms*. 2018. Cham: Springer International Publishing.
5. Qin, W., et al., *Sources and migration of heavy metals in a karst water system under the threats of an abandoned Pb–Zn mine, Southwest China.* Environmental Pollution, 2021. **277**: p. 116774.
6. Ghosh, S., et al., *Chapter 10 - Microbial biosorbents for heavy metal removal*, in *New Trends in Removal of Heavy Metals from Industrial Wastewater*, M.P. Shah, S. Rodriguez Couto, and V. Kumar, Editors. 2021, Elsevier. p. 213-262.
7. Adesiyan, I., et al., *Concentrations and Human Health Risk of Heavy Metals in Rivers in Southwest Nigeria.* Journal of Health and Pollution, 2018. **8**: p. 180907.
8. Vishnu, D., et al., *Chapter 8 - Removal of heavy metals from mine waters by natural zeolites*, in *New Trends in Removal of Heavy Metals from Industrial Wastewater*, M.P. Shah, S. Rodriguez Couto, and V. Kumar, Editors. 2021, Elsevier. p. 161-175.
9. Ansari, M.I. and A. Malik, *Biosorption of nickel and cadmium by metal resistant bacterial isolates from agricultural soil irrigated with industrial wastewater.* Bioresource Technology, 2007. **98**(16): p. 3149-3153.
10. Cristani, M., et al., *Possible use of Serratia marcescens in toxic metal biosorption (removal).* Environmental Science and Pollution Research, 2012. **19**(1): p. 161-168.
11. Wu, H., et al., *Cd-Resistant Strains of B. cereus S5 with Endurance Capacity and Their Capacities for Cadmium Removal from Cadmium-Polluted Water.* PloS one, 2016. **11**: p. e0151479.

12. Ozdemir, S., et al., *Simultaneous preconcentrations of Co2+, Cr6+, Hg2+ and Pb2+ ions by Bacillus altitudinis immobilized nanodiamond prior to their determinations in food samples by ICP-OES.* Food Chemistry, 2017. **215**: p. 447-453.

13. Tajer-Mohammad-Ghazvini, P., et al., *Cobalt separation by Alphaproteobacterium MTB-KTN90: magnetotactic bacteria in bioremediation.* Bioprocess and Biosystems Engineering, 2016. **39**(12): p. 1899-1911.

14. Yalçın, M.S., S. Özdemir, and E. Kılınç, *Preconcentrations of Ni(II) and Co(II) by using immobilized thermophilic Geobacillus stearothermophilus SO-20 before ICP-OES determinations.* Food Chemistry, 2018. **266**: p. 126-132.

15. Zárate, A., et al., *Burkholderia tropica as a Potential Microalgal Growth-Promoting Bacterium in the Biosorption of Mercury from Aqueous Solutions.* Journal of microbiology and biotechnology, 2017. **27**.

16. Mondal, P., C.B. Majumder, and B. Mohanty, *Treatment of arsenic contaminated water in a batch reactor by using Ralstonia eutropha MTCC 2487 and granular activated carbon.* Journal of Hazardous Materials, 2008. **153**(1): p. 588-599.

17. Prasad, K.S., et al., *Biosorption of arsenite (As+3) and arsenate (As+5) from aqueous solution by Arthrobacter sp biomass.* Environmental technology, 2013. **34**: p. 2701-8.

18. Podder, M.S. and C.B. Majumder, *Corynebacterium glutamicum MTCC 2745 immobilized on granular activated carbon/MnFe2O4 composite: A novel biosorbent for removal of As(III) and As(V) ions.* Spectrochimica Acta Part A: Molecular and Biomolecular Spectroscopy, 2016. **168**: p. 159-179.

19. Asadi Haris, S., et al., *Arsenic biosorption using pretreated biomass of psychrotolerant Yersinia sp. strain SOM-12D3 isolated from Svalbard, Arctic.* Environmental Science and Pollution Research, 2018. **25**(28): p. 27959-27970.

20. Sherlala, A.I.A., et al., *A review of the applications of organo-functionalized magnetic graphene oxide nanocomposites for heavy metal adsorption.* Chemosphere, 2018. **193**: p. 1004-1017.
